# An updated checklist of freshwater rotifers in Thailand: Taxonomy, diversity and distribution

**DOI:** 10.3897/BDJ.13.e155363

**Published:** 2025-07-11

**Authors:** Rapeepan Jaturapruek, Supiyanit Maiphae

**Affiliations:** 1 Animal Systematics and Ecology Research Unit, Department of Zoology, Faculty of Science, Kasetsart University, Bangkok, Thailand Animal Systematics and Ecology Research Unit, Department of Zoology, Faculty of Science, Kasetsart University Bangkok Thailand; 2 Biodiversity Center Kasetsart University (BDCKU), Bangkok, Thailand Biodiversity Center Kasetsart University (BDCKU) Bangkok Thailand

**Keywords:** bdelloid rotifers, taxonomy, biodiversity, distribution, Oriental region

## Abstract

**Background:**

The Rotifera is the most diverse group of freshwater zooplankton. In Thailand, the research on rotifers began in 1907. Following extensive studies on rotifers in Thai water bodies, the first checklist was published in 2013, with approximately 398 species. However, the taxonomy and diversity of bdelloid rotifers, a major group of freshwater rotifers, have only been intensively studied since 2016. Therefore, we provide an updated and comprehensive checklist of freshwater rotifers in Thailand, including nomenclatural updates, species diversity, and geographical distribution.

**New information:**

An updated checklist of rotifer fauna from inland aquatic habitats in Thailand—a high-diversity hotspot in Southeast Asia—is presented based on published records. This checklist includes revised nomenclature and updated species distributions. A total of 409 valid recorded species reflects relatively high diversity. However, estimators suggest that more species are likely to be discovered with further research. Planktonic monogononts are relatively well studied, whereas sessile monogononts and bdelloids require more attention. Additionally, large research gaps remain in the western and eastern regions. Each habitat type appears to support a distinct rotifer community, as indicated by generally low similarity values (Jaccard index < 0.53). Thus, habitats with unique characteristics, such as cave pools and limno-terrestrial habitats, warrant further investigation. Since the taxonomic status and species diversity have been consolidated, the geographical distribution is discussed.

## Introduction

Taxonomic studies are essential for understanding species richness, as they provide crucial information on biodiversity, global species distribution patterns, and environmental changes related to climate or global trade. The phylum Rotifera is a diverse group of zooplankton found in nearly all freshwater and semi-aquatic habitats (Wallace 2002, Segers 2008). This phylum comprises two major groups, Monogononta and Bdelloidea, whereas Seisonacea represents a small group of epizoic marine species (Segers 2007, Fontaneto et al. 2008, Segers 2008). Rotifers are highly diverse and widespread in continental aquatic environments (Fontaneto and De Smet 2015). They play a crucial role in aquatic food webs as primary consumers of phytoplankton (Allan 1976, Wallace et al. 2015) and serve as effective biological indicators for assessing water quality (Wallace et al. 2006).

Research on rotifers in Thailand began in 1907 with the work of Weber. Since then, increasing attention has been given to this group of microorganisms, particularly monogonont rotifers, resulting in a growing number of published studies. Numerous studies have focused on taxonomy, species diversity, and distribution across various habitat types (Weber 1907, De Ridder 1971, Koste 1975, Boonsom 1984, Sanoamuang et al. 1995, Sanoamuang 1996, Pholpunthin 1997, Sanoamuang and Segers 1997, Segers and Pholpunthin 1997, Pholpunthin and Chittapun 1998, Sanoamuang 1998, Chittapun et al. 1999, Sanoamuang and Savatenalinton 1999, Chittapun and Pholpunthin 2001, Sanoamuang and Savatenalinton 2001, Segers and Chittapun 2001, Chittapun et al. 2002, Chittapun et al. 2003, Segers et al. 2004, Athibai et al. 2005, Savatenalinton and Segers 2005, Jithlang and Wongrat 2006, Teeramaethee et al. 2006, Chittapun et al. 2007, Sanoamuang 2007, Chittapun et al. 2009, Segers and Savatenalinton 2010, Meksuwan et al. 2011, Athibai et al. 2013, Meksuwan et al. 2013, Sa-ardrit et al. 2017, Meksuwan et al. 2018, Jantawong and Maiphae 2020, Plangklang and Athibai 2021, Jantawong et al. 2023, Maiphae et al. 2023), while fewer studies have explored their ecology such as the recovery of rotifers from resting eggs (Chittapun et al. 2005, Athibai and Sanoamuang 2008, Chittapun 2011).

Following extensive studies on rotifers in Thai water bodies, the first checklist was published in 2013, documenting approximately 398 species (Sa-Ardrit et al. 2013). However, the taxonomy and diversity of freshwater bdelloid rotifers, a distinct group within the phylum Rotifera, have only been intensively studied since 2016 (Jaturapruek et al. 2018, Jaturapruek et al. 2020, Jaturapruek et al. 2021). As a result, numerous additional species have been discovered in recent years, and the taxonomic status of several species has been revised. In this study, we present an updated and comprehensive checklist of freshwater rotifers in Thailand, incorporating recent taxonomic insights, nomenclatural updates, species diversity, and geographical distribution.

## Materials and methods

### Data collection

In this study, a checklist of freshwater rotifer species in Thailand was compiled from 41 published research papers (Table 1). Updated taxonomic names, based on the List of Available Names in Zoology (LAN) (Jersabek et al. 2018), were used consistently in all analyses. The occurrence of rotifers in Thailand was categorized into six geographical regions—northern, northeastern, central, western, eastern, and southern—based on mountain ranges and topography (Setapan 1999). Information on biogeographical distribution was primarily obtained from Segers (2007) and supplemented with additional literature, as summarized in Table 2. Species occurrences were classified into eight major biogeographical regions: Palearctic, Afrotropical, Oriental, Nearctic, Neotropical, Australian, Pacific, and Antarctic, following Segers (2007). The present dataset has been published in GBIF (DOI: https://doi.org/10.15468/jmk6jp).

Habitats were categorized into 13 types based on their characteristics, following Keddy (2010). A ‘canal’ was defined as a waterway channel or artificial waterway that is smaller and shallower than a river. Some standing-water canals were covered with algae. A ‘dam’ was defined as a wall built across a river to stop its flow and collect water. A ‘fish field’ was defined as a fish farm pond. An ‘irrigation tank’ was defined as an artificial reservoir of any size, often with an earthen bund constructed across a long slope to collect and store surface water from the upstream catchment. A ‘lake’ was considered a large natural area (at least 80,000 m²) filled with water, apart from rivers that serve to drain the lake. A ‘marsh’ was defined as a wetland that is permanently wet and soft, dominated by herbaceous rather than woody plant species. A ‘peat swamp’ was defined as a wetland where waterlogged soil prevents dead leaves and wood from fully decomposing; over time, this creates a thick layer of acidic peat. A ‘pond’ was defined as a standing water body, either natural or artificial, that is smaller than a lake (less than 80,000 m²) and may be covered by vegetation. A ‘reservoir’ was defined as a large natural or artificial water body used as a source of water supply. A ‘rice field’ was defined as a flooded parcel of land used for growing rice crops. A ‘river’ was defined as a natural flowing watercourse that moves toward an ocean, sea, lake, or another river, usually consisting of freshwater. A ‘swamp’ was defined as a forested wetland that occurs near large rivers or lakes and is critically dependent on natural water level fluctuations; some swamps were covered by dense aquatic vegetation. A ‘temporary pond’ was defined as a shallow water body that undergoes a periodic cycle of flooding and drought.

### Data analysis

Sampling effort can strongly influence species numbers; therefore, three species richness estimators—Jackknife 1, Jackknife 2, and Bootstrap—were used to estimate the expected diversity of rotifers. These estimations were performed using a species accumulation curve in EstimateS (Colwell 2013). To compare rotifer species composition across different regions and habitat types, the Jaccard similarity index was calculated using Microsoft Excel 2016.

## Identification Keys

### Keys to species of freshwater bdelloid rotifers in Thailand

**Table:** 

1	Stomach with faecal pellets, usually several teeth	2
–	Stomach without faecal pellets, usually few teeth	4
2	Ring present on the trochus, 6–10 teeth	*Otostephanosdonneri*
–	Ring absent on the trochus, mostly >2 teeth	3
3	Lower lip inconspicuous, head without keel, dorsal antenna extremely long and cylindrical	*Habrotrochaampulla*
–	Lower lip distinguished, head with keel, dorsal antenna long	*Habrotrochaangusticollis*
4	Oviparous, 4 toes, integument smooth, usually 2 teeth, eyes on the brain	*Philodinamegalotrocha*
–	Viviparous	5
5	Eyes often on the rostrum, 3 toes	6
–	No eyes on the rostrum, 4 toes	13
6	Foot length longer than body length 2 times	7
–	Foot length shorter than body length	8
7	Trunk oval shape, spurs without joint	*Rotariaovata*
–	Trunk cylindrical shape, spurs with two joints	*Rotarianeptunia*
8	Foot length 1-1/2 of body length	9
–	Foot length 1/4 of body length	11
9	Three toes unequal	*Rotariamegarostris*
–	Three toes equal	10
10	Trunk clearly separated from foot segments	*Rotariamacrura*
–	Trunk unclearly separated from foot segments	*Rotariarotatoria*
11	Spurs short, stout and without joints	*Rotariamento*
–	Spurs long with two joints	12
12	Spurs slender and sharp tips, trunk transparent	*Rotarianeptunoida*
–	Spurs thick and blunt tips, trunk with detritus	*Rotariatardigrada*
13	Trunk with pointed or blunt spines	*Dissotrochaaculeata*
–	Trunk without spines or thorns	*Dissotrochamacrostyla*

This key has been adapted from Ricci and Melone (2000).

## Analysis

### Taxonomy and species diversity

The taxonomic status of rotifers in Thailand has been revised. Meksuwan et al. (2011) proposed a new genus of sessile rotifer, *Lacinularoides*, which has morphological characteristics very similar to *Lacinularia* Schweigger, 1820, except for differences in the corona. *Lacinularia* has a heart-shaped corona, while *Lacinularoides* possesses a large pair of ventral lobes, a pair of small lobes, and three dorsal lobes. Moreover, *Ptyguranoodti* (Koste, 1972) was reallocated from the genus *Floscularia* Cuvier, 1798, based on observations of living specimens. Previously, this species was known only from preserved and contracted specimens from the Amazon region (Meksuwan et al. 2011). In addition, since 2011, five species—*Collothecaorchidacea*, *Ptygurathalenoiensis*, *Limniaslenis*, *L.novemceras*, and *Rotariamegarostris*—have been described as new species.

Therefore, a total of 409 freshwater rotifer species, belonging to 68 genera and 26 families, have been recorded in 41 published studies in Thailand. Seventeen species—*Collothecaferox*, *C.orchidacea*, *Limniaslenis*, *L.novemceras*, *Mytilinatrigona*, *Otostephanosdonneri*, *Philodinamegalotrocha*, *Platyiasleloupi*, *Rotariamacrura*, *R.megarostris*, *R.mento*, *R.neptunia*, *R.neptunoida*, *R.ovata*, *R.rotatoria*, *R.tardigrada*, and *Testudinellaincisa*—have been updated from the previous record by Sa-Ardrit et al. (2013). The dominant group in the Thai rotifer fauna is Monogononta, with 395 species, while Bdelloidea comprises only 14 species. Of these, 351 species are classified as planktonic and 58 species as sessile rotifers, based on their mode of living. Given the increasing number of studies on bdelloid rotifers and the growing number of species reported from freshwater habitats in Thailand, a key to species is also provided in this study to facilitate future identification and research.

**Figure 1.**
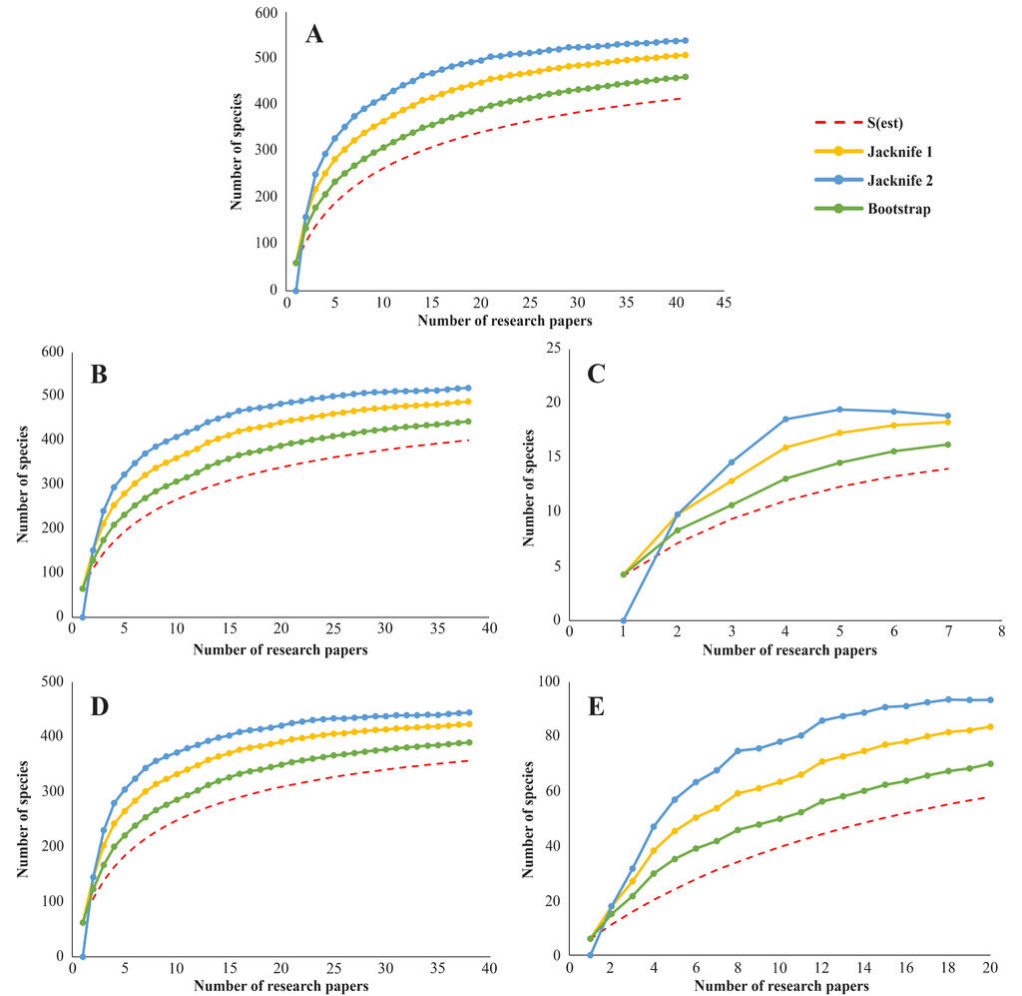
Species accumulation curves of freshwater rotifers in Thailand. (A) Overall potential richness; (B) Monogononta; (C) Bdelloidea; (D) Planktonic rotifers; (E) Sessile rotifers.

The species accumulation curve for rotifer species richness in Thailand indicated that the results of all three estimators (Jackknife 1, Jackknife 2, and Bootstrap) exceeded the observed number of species. Overall, the sampling effort (measured by the number of published studies) estimated the potential rotifer richness in Thailand to range from 460.4 species (Bootstrap) to 507.7 species (Jackknife 1) and 538.7 species (Jackknife 2) (Fig. 1A). The Jackknife 2 estimator provided the highest maximum value in all cases, including Monogononta (519.5 species, 38 research papers), Bdelloidea (18.9 species, 7 research papers), planktonic rotifers (444.3 species, 38 research papers), and sessile rotifers (93.5 species, 20 research papers) (Fig. 1B–E).

The most diverse family was Lecanidae, with 96 recorded species (23.47%), followed by Lepadellidae (44 species, 10.76%), Brachionidae (43 species, 10.51%), Flosculariidae (38 species, 9.29%), and Trichocercidae (35 species, 8.56%). The most diverse genus was *Lecane* (96 species), followed by *Trichocerca* (35 species), *Lepadella* (30 species), and *Brachionus* (28 species).

### Regional distribution in Thailand

**Figure 2.**
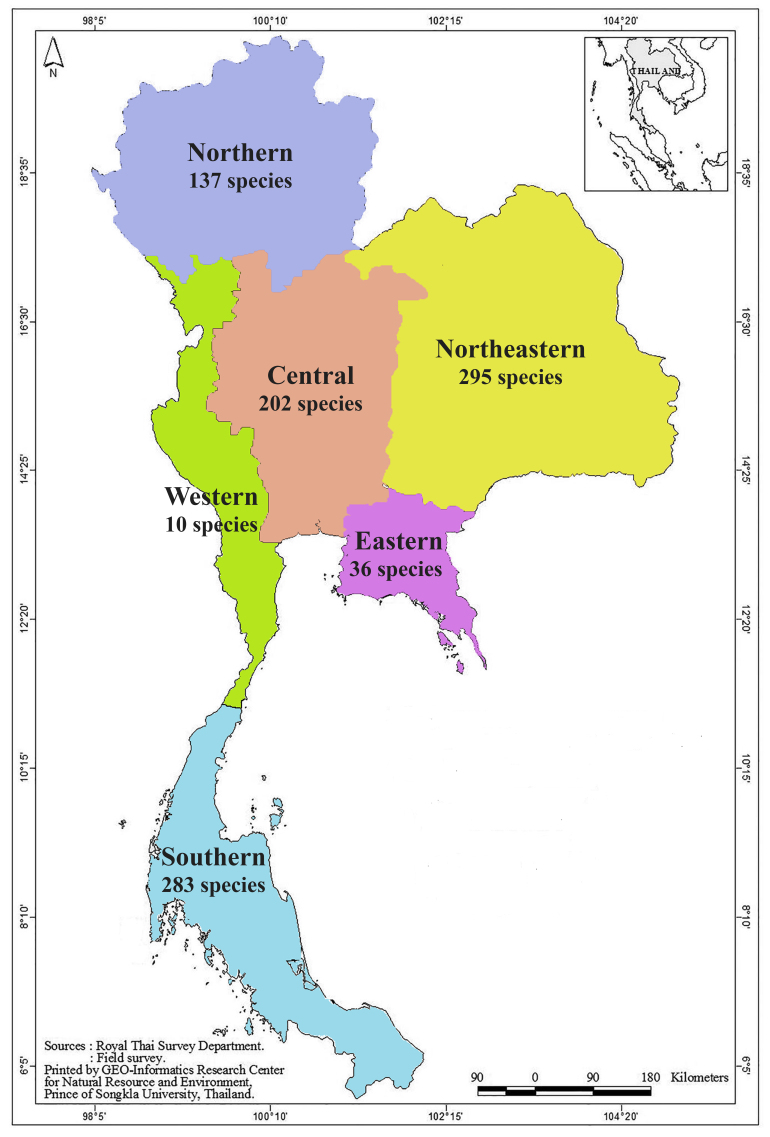
The species richness of rotifers in each region of Thailand.

**Figure 3.**
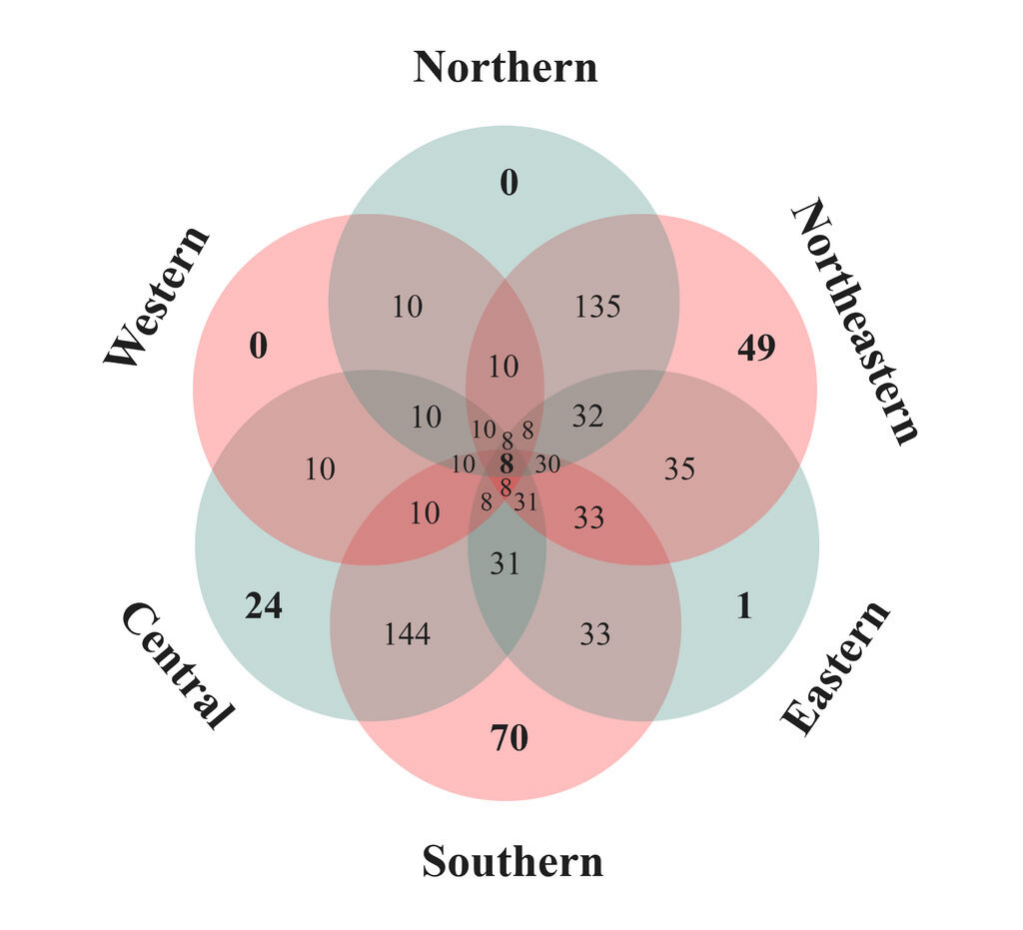
The circle Venn diagram indicates the number of rotifers limited to each region and shared between regions in Thailand.

With respect to regional distribution of rotifers across the six regions of Thailand, the northeastern region exhibited the highest rotifer richness (295 species), followed by the southern (283 species), central (202 species), northern (137 species), eastern (36 species), and western regions (10 species) (Fig. 2). Only eight species—*Brachionuscalyciflorus*, *B.forficula*, *Keratellatropica*, *Rotariamegarostris*, *R.mento*, *R.neptunia*, *R.ovata*, and *R.tardigrada*—were found in all regions of Thailand, while many species were restricted to a single region. For example, *L.armata* was found only in the eastern region. Additionally, 24 species were found exclusively in the central region, 49 species in the northeastern region, and 70 species in the southern region. Notably, no rotifer species was found to be restricted to the northern and western regions (Fig. 3).

The Jaccard similarity index for species composition across different regions in Thailand ranged from 0.03 to 0.53, indicating low to intermediate similarity between regions. The northeastern and southern regions exhibited the highest similarity, with more than 50% of species shared, followed by the northeastern and central regions (48%), and the northern and northeastern regions (45%). In contrast, the northeastern and western regions showed the lowest similarity, with only 3% of species shared (Table 3).

### Ecological distribution

**Figure 4.**
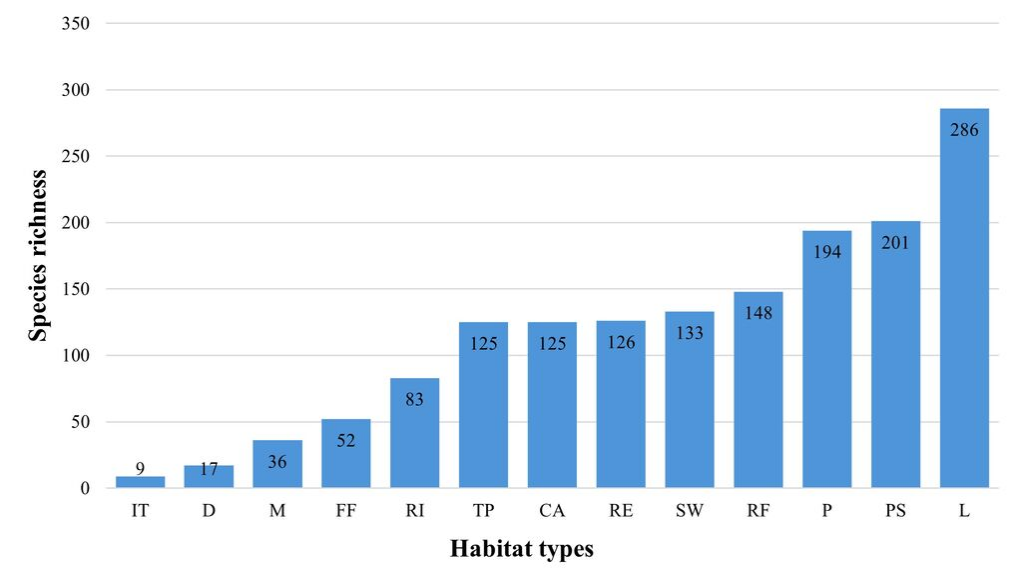
Species richness of rotifers in across habitat types. Habitat type abbreviations are as defined in Table 2.

Among the various freshwater habitats surveyed in Thailand, the highest rotifer species richness was recorded in lakes (286 species), followed by peat swamps (201 species), ponds (194 species), rice fields (148 species), and swamps (133 species). In contrast, irrigation tanks exhibited the lowest richness, with only nine recorded species (Fig. 4).

Additionally, the Jaccard similarity index for rotifer species composition across different habitat types ranged from 0.00 to 0.53, with most habitats sharing less than 50% similarity. The highest similarity was observed between canals and ponds, as well as rice fields and temporary ponds, with 53% of species shared, followed by canals and rice fields, with 51%. In contrast, dams and irrigation tanks showed no similarity, indicating completely distinct species compositions between these two habitats (Table 4).

A total of 32 rotifer species were recorded across a wide range of habitat types (≥10). Among these, only five species—*Brachionuscalyciflorus*, *Lecanebulla*, *L.curvicornis*, *L.papuana*, and *L.quadridentata*—were present in nearly all habitat types (12). In contrast, 127 species were found in only a single habitat type. For example, *Wolgaspinifera* was found only in temporary ponds. Two species—*Limniasnovemceras* and *Lecaneopias* were found only in rivers, whereas *Otostephanosdonneri* and *Rotariamacrura* were recorded exclusively in swamps. Three species—*Cephalodellatenuiseta*, *Mytilinatrigona*, and *Testudinellaincisa*—were restricted to rice fields. Additionally, five species—*Brachionusleydigii*, *Dicranophorusrobustus*, *Lecanelamellata*, *L.lauterborni*, and *Lepadellaelliptica*—were exclusive to fish ponds. Moreover, 10, 12, 34, and 58 species were found only in ponds, reservoirs, peat swamps, and lakes, respectively. Although irrigation tanks had the lowest species richness, all species recorded in this habitat were also found in other habitat types.

Although most Thai rotifer species are widespread or cosmopolitan, several species are endemic to the Oriental region or Thailand. Of all recorded species, 28 (6.85%) were identified as Oriental endemics, while 11 species (2.69%)—*Collothecaorchidacea*, *Encentrumpornsilpi*, *Keratellataksinensis*, *Lecanejunki*, *L.lungae*, *L.martensi*, *L.micrognatha*, *L.segersi*, *Limniaslenis*, *L.novemceras*, and *Rotariamegarostris*—were endemic or regionally restricted to Thailand. In contrast, 16 species were found across all eight biogeographical regions.

## Discussion

### Species diversity

The number of freshwater rotifer species recorded in Thailand has continued to increase due to more intensive research across diverse microhabitats. To date, approximately 409 valid rotifer species have been recorded from Thailand, representing about 20.14% of the total species within the phylum Rotifera (Segers 2008). The species richness of Thai monogonont rotifers is higher than that of other Southeast Asian countries, including Cambodia (306 species) (Sor et al. 2015), Vietnam (227 species) (Trinh-Dang et al. 2019), Malaysia (220 species) (Segers 2004), Laos (135 species) (Segers and Sanoamuang 2007), the Philippines (115 species) (Tuyor and Segers 1999), and Myanmar (100 species) (Koste and Tobias 1990). The differences in recorded species richness among these Southeast Asian countries are likely influenced more by the extent of research efforts rather than actual species diversity. Many of these countries possess a high diversity of aquatic habitats, which suggests the potential for greater species richness. If more studies were conducted in these regions, it is likely that additional species would be discovered, further increasing the recorded diversity.

Records of freshwater bdelloid rotifers from Southeast Asia are notably scarce. In Thailand, only 14 species have been recorded, compared to 58 species reported from the Oriental region (Segers 2008). However, bdelloid species richness is higher in Malaysia (30 species) (Fontaneto and Ricci 2004) and Indonesia (30 species) (Hauer 1937, Hauer 1938, Bartoš 1963, Koste 1988) than in Thailand, whereas Myanmar (5 species) (Koste and Tobias 1990) and Vietnam (4 species) (Shirota 1966) have reported lower diversity. One of the main challenges in studying bdelloid rotifers is the need to examine key morphological characteristics while the organisms are alive. Moreover, past research in Thailand has primarily focused on the systematics of only a few genera such as *Rotaria* (Jaturapruek et al. 2018, Jaturapruek et al. 2020, Jaturapruek et al. 2021), leaving many others largely unexplored. Additionally, their ecology and evolutionary relationships remain poorly understood (Wallace et al. 2006, Segers 2008). This paper focuses on freshwater habitats; however, further research should target underexplored microhabitats, particularly limno-terrestrial environments (Donner 1965, Ricci 1987, Ricci 2001), which are closely linked to bdelloids' anhydrobiotic capabilities (Ricci 1998, Ricci and Melone 2000, Ricci 2001) and unique behavioral adaptations (Ricci and Melone 2000, Segers 2008). Although some studies have been conducted in limno-terrestrial habitats, 34 bdelloid rotifer species have so far been found in bryophytes (Jattupan et al. 2024, Pokpongmongkol et al. 2025). Addressing these knowledge gaps would provide valuable insights into the diversity and ecological roles of this fascinating group of rotifers.

The northeastern region of Thailand exhibits the highest rotifer species richness, with 295 species recorded. This region is not only the largest in the country but also the most extensively studied (Sa-Ardrit et al. 2013). Similarly, the southern region has the second-highest rotifer diversity (283 species), likely due to its wide variety of freshwater habitats and intensive research efforts. Additionally, most Ramsar sites in Thailand are located in the northeastern (Kud-Thing Lake and Khong Long Swamp) and southern (Thale-Noi Lake and To Daeng Peat Swamp) regions (Ramsar Sites Information Service 2023), contributing to the high diversity of not only rotifers but also other groups of zooplankton (Choedchim and Maiphae 2023). However, despite both regions having a high number of species, their species composition is only 53% similar. Of these, 70 species are restricted to the southern region, while 49 species are exclusive to the northeastern region. This suggests that even though they share similar types of aquatic habitats, the specific characteristics of each water body—particularly environmental factors such as temperature, pH, turbidity, trophic state (Phan et al. 2021), and dissolved oxygen (Jaturapruek et al. 2020)—as well as the level of habitat complexity (Fontaneto et al. 2006, Fontaneto et al. 2011, Kuczyńska-Kippen 2018, Jaturapruek et al. 2020) likely play a crucial role in shaping regional rotifer assemblages. Aquatic vegetation creates a more diverse environment, provides a rich food source for many zooplankton species, and serves as an effective refuge from predators (Stansfield et al. 1997, Choedchim et al. 2017).

Furthermore, while rotifer research in Thailand appears comprehensive, species richness estimators indicate that the number of existing studies is still inadequate, suggesting that many species remain undiscovered. To address this gap, further intensive research is particularly needed in the eastern and western regions, where relatively few studies have been conducted despite the presence of diverse and unique freshwater habitats. For instance, the eastern region includes coastal freshwater systems, which may be influenced by seawater during certain seasons, while the western region is characterized by numerous stream branches. Expanding studies in these underexplored areas will contribute to a more complete dataset, providing a clearer representation of the true diversity of freshwater rotifers in Thailand.

### Ecological distribution

Lakes are ecologically heterogeneous environments that support the highest rotifer diversity among freshwater habitat types in Thailand. A total of 286 species have been recorded in these habitats, accounting for approximately 69.93% of the known rotifer species in Thailand. This is consistent with findings on the high diversity of other zooplankton groups, such as cladocerans, which are also predominantly distributed in this habitat type (Choedchim and Maiphae 2023). Species diversity in Kud-Thing Lake, Bueng Kan Province (Northeastern Thailand), is remarkable, with 183 recorded species (Sanoamuang and Savatenalinton 2001), followed by Thale-Noi Lake, Phatthalung Province (Southern Thailand), with at least 106 species (Segers and Pholpunthin 1997, Meksuwan et al. 2011). These two lakes are Ramsar sites in Thailand. Kud-Thing Lake is a large natural lake that receives sediment from the Mekong River, while Thale-Noi Lake is connected to Songkhla Lake (Ramsar Sites Information Service 2023). These geographical features contribute to the structural complexity of the lakes, enabling organisms to occupy various microhabitats and ecological niches. Consequently, these lakes are among the biodiversity hotspots for rotifers in Southeast Asia. In contrast, irrigation tanks exhibited the lowest rotifer species richness, likely due to the absence of habitat complexity, which restricts the diversity of ecological niches available. Additionally, short water retention times and frequent fluctuations in water levels may further impede the establishment and stability of planktonic communities in these artificial systems.

Similar habitat structures were found to result in similar rotifer compositions. Rice fields showed a high similarity in rotifer communities with temporary ponds, as both habitats undergo continuous water level fluctuations throughout the rice-growing cycle. This pattern is comparable to temporary ponds, which also experience alternating wet and dry periods. Similarly, canals and ponds—both natural or artificial standing water habitats, often covered by vegetation or algae—exhibited high similarity in rotifer composition. In contrast, certain habitat types, such as dams and irrigation tanks, have unique structural characteristics, resulting in low similarity with other habitats. However, all species recorded in these habitats were also found in other habitat types.

We highlight that, despite intensive rotifer research in Thailand, some habitats remain understudied. For instance, cave pools, which are characterized by low light levels and often low oxygen concentrations, require organisms to develop morphological and behavioral adaptations. While these habitats have been well studied for other zooplankton groups, such as copepods—where a high number of species have been recorded in these extreme environments (i.e. Watiroyram et al. 2015, Watiroyram and Brancelj 2016, Watiroyram et al. 2017, Sanoamuang and Watiroyram 2021, Watiroyram et al. 2021)—there is a lack of information on rotifers in these habitats. Although rotifers in limno-terrestrial habitats, such as mosses, lichens, and soil, have been studied in Thailand (Jattupan et al. 2024, Pokpongmongkol et al. 2025), further research is still needed.

In addition, approximately 30% of the total recorded species were restricted to a single habitat type, whereas only 1% were widely distributed across up to 12 habitat types. *Lecanebulla* and *L.papuana* are widely distributed and can be found in diverse habitats (Maiphae et al. 2023). From a zoogeographical perspective, these species are considered cosmopolitan (Segers 2007).

Moreover, *Brachionuscalyciflorus* exhibits morphological adaptations in response to predation pressure and pollution. Under high predation pressure, it develops stable, long posterior lateral spines and a relatively small body size. Additionally, in polluted environments, it adapts by reducing body size and producing larger eggs (Xue et al. 2017). Due to this ability, *B.calyciflorus* occurs in nearly all habitat types (12). In contrast, some species, such as *B.leydigii*, exhibit significant habitat specialization, preferring environments with high chlorophyll a and total phosphorus concentrations (Le Coz et al. 2018), as evidenced by its exclusivity to fish fields.

### Biogeographical distribution

Currently, approximately 64% of Thai rotifer fauna consists of cosmopolitan species, defined as those present in at least five of the eight biogeographical regions of the world (Segers 2008). Only 16 species are distributed across all eight biogeographical regions. However, a considerable number of species are endemic to the Oriental region (28 species) and Thailand (11 species). Due to the discovery of newly reported species and the taxonomic revision of certain rotifer groups, the number of Thai endemic species has decreased compared to the previous study by Sa-Ardrit et al. (2013). Here, we provide an updated distribution of some taxa that were previously recorded as Thai endemics. *Colurellapsammophila* is now known to be restricted to southern Thailand (Segers and Chittapun 2001, Chittapun et al. 2007) and China (Wei and Xu 2014, Wei et al. 2019). *Brachionussrisumonae*, *Lecaneisanensis*, and *L.niwati* are exclusively found in northeastern Thailand (Sanoamuang and Savatenalinton 2001, Segers et al. 2004, Segers and Savatenalinton 2010) and India (Sharma et al. 2017, Sharma and Sharma 2019, Sharma and Sharma 2021). *L.kunthuleensis* is known from southern Thailand (Chittapun et al. 2003, Segers and Savatenalinton 2010), China (Huang et al. 2017), and Vietnam (Duong et al. 2021). Additionally, *L.superaculeata* has been reported from Thailand, India (Sharma et al. 2017, Sharma and Sharma 2019, Sharma and Sharma 2021), Vietnam (Duong et al. 2021), and Cambodia (Sor et al. 2015). *L.acanthinula* has extended its range beyond the traditional boundaries of the Oriental region (Segers 2001, Sharma and Sharma 2021), now including both northeastern (Sanoamuang et al. 1995, Sanoamuang and Segers 1997) and southern Thailand (Segers and Chittapun 2001, Chittapun et al. 2007), as well as the Palearctic region, with records from Turkey (Bozkurt 2017). This extension highlights the ongoing impact of sampling effort on rotifer biodiversity—the so-called rotiferologist effect—persisting even in well-studied countries (Fontaneto et al. 2012, Jaturapruek et al. 2018, Jaturapruek et al. 2020).

## Figures and Tables

**Table 1.** References used for data collection.

1 = De Ridder (1971)	22 = Jithlang and Wongrat (2006)
2 = Koste (1975)	23 = Teeramaethee et al. (2006)
3 = Boonsom (1984)	24 = Chittapun et al. (2007)
4 = Sanoamuang et al. (1995)	25 = Sanoamuang (2007)
5 = Sanoamuang (1996)	26 = Athibai and Sanoamuang (2008)
6 = Pholpunthin (1997)	27 = Chittapun et al. (2009)
7 = Sanoamuang and Segers (1997)	28 = Segers and Savatenalinton (2010)
8 = Segers and Pholpunthin (1997)	29 = Chittapun (2011)
9 = Pholpunthin and Chittapun (1998)	30 = Meksuwan et al. (2011)
10 = Sanoamuang (1998)	31 = Athibai et al. (2013)
11 = Chittapun et al. (1999)	32 = Meksuwan et al. (2013)
12 = Sanoamuang and Savatenalinton (1999)	33 = Sa-ardrit et al. (2017)
13 = Chittapun and Pholpunthin (2001)	34 = Jaturapruek et al. (2018)
14 = Sanoamuang and Savatenalinton (2001)	35 = Meksuwan et al. (2018)
15 = Segers and Chittapun (2001)	36 = Jantawong and Maiphae (2020)
16 = Chittapun et al. (2002)	37 = Jaturapruek et al. (2020)
17 = Chittapun et al. (2003)	38 = Jaturapruek et al. (2021)
18 = Segers et al. (2004)	39 = Plangklang and Athibai (2021)
19 = Athibai et al. (2005)	40 = Maiphae et al. (2023)
20 = Chittapun et al. (2005)	41 = Jantawong et al. (2023)
21 = Savatenalinton and Segers (2005)	

**Table 2.** Checklist of freshwater rotifers in Thailand. Habitat types are abbreviated as follows: CA = canal, D = dam, FF = fish field, IT = irrigation tank, L = lake, M = marsh, P = pond, PS = peat swamp, RE = reservoir, RF = rice field, RI = river, SW = swamp, TP = temporary pond. Distributions are represented as N = northern, NE = northeastern, C = central, E = eastern, W = western, S = southern, PAL = Palearctic, AFR = Afrotropical, ORI = Oriental, NEA = Nearctic, NEO = Neotropical, AUS = Australian, PAC = Pacific, ANT = Antarctic. References correspond to those listed in Table 1. * = Updated record from Sa-Ardrit et al. (2013).

	**Species**	**Habitat**	**Distribution in Thailand**	**Biogeographical distribution**	**References**	**Remark**
**Bdelloidea**						
	**Family Habrotrochidae**					
1	*Habrotrochaampulla* Murray, 1911	no information	C	AFR, NEA, NEO, ORI, PAL	2	
2	*Habrotrochaangusticollis* (Murray, 1905)	no information	C	AFR, ANT, AUS, NEA, NEO, ORI, PAL	2	
3	*Otostephanosdonneri* Bartoš, 1959*	SW	S	AUS, ORI, PAL	34	
	**Family Philodinidae**					
4	*Dissotrochaaculeata* (Ehrenberg, 1830)	L, PS, SW	N, NE, C, E, S	AFR, AUS, NEA, NEO, ORI, PAL	2, 13, 16, 24, 34	
5	*Dissotrochamacrostyla* (Ehrenberg, 1838)	L, SW	N, NE, C, S	AFR, AUS, NEA, NEO, ORI, PAL	2, 34	
6	*Philodinamegalotrocha* Ehrenberg, 1832*	P, SW	NE, S	AFR, AUS, NEA, NEO, ORI, PAL	34	
7	*Rotariamacrura* (Ehrenberg, 1832)*	SW	N, C	AFR, AUS, NEA, NEO, ORI, PAL	37	
8	*Rotariamegarostris* Jaturapruek, Fontaneto, Meksuwan, Pholpunthin & Maiphae, 2018*	CA, L, P, PS, SW	N, NE, C, E, W, S	ORI (Endemic to Thailand)	34, 37	
9	*Rotariamento* (Anderson, 1889)*	CA, L, M, P, RF, SW	N, NE, C, E, W, S	NEA, ORI, PAL	34, 37	
10	*Rotarianeptunia* (Ehrenberg, 1830)*	CA, L, P, RF, RI, SW	N, NE, C, E, W, S	AFR, AUS, NEA, NEO, ORI, PAL	34, 37, 38	
11	*Rotarianeptunoida* Harring, 1913*	CA, M, P, PS, RF, SW	N, NE, C, W, S	AFR, AUS, NEA, ORI, PAL	34, 37	
12	*Rotariaovata* (Anderson, 1889)*	P, PS, SW	N, NE, C, E, W, S	ORI (Known from India and Thailand)	34, 37	
13	*Rotariarotatoria* (Pallas, 1766)*	CA, L, M, P, PS, SW	N, NE, C, W, S	AFR, AUS, NEA, NEO, ORI, PAL	34, 37, 38	
14	*Rotariatardigrada* (Ehrenberg, 1830)*	CA, L, M, P, RI, SW	N, NE, C, E, W, S	AFR, AUS, NEA, NEO, ORI, PAL	34, 37, 38	
**Monogononta**						
	**Family Asplanchnidae**					
15	*Asplanchnabrightwellii* Gosse, 1850	FF, L, P, RE, SW	NE, C	AFR, AUS, NEA, NEO, ORI, PAC, PAL	1, 3, 4, 14, 18, 21, 22, 23	Incl.: *Asplanchnabrightwelli* Gosse, 1850 (Ref.1,4,14,18,22)
16	*Asplanchnapriodonta* Gosse, 1850	CA, L, P, RE, RI, SW, TP	N, NE, C	AFR, AUS, NEA, NEO, ORI, PAL	3, 4, 5, 10, 14, 18, 21, 22, 23, 25	
17	*Asplanchnasieboldii* (Leydig, 1854)	CA, L, P, PS, RF, RI, TP	N, NE, C, S	AFR, AUS, NEA, NEO, ORI, PAL	4, 10, 18, 24, 25, 27, 29, 40	Incl.: *Asplanchnasieboldi* (Leydig, 1854) (Ref.4,10,18,24)
18	*Asplanchnatropica* Koste & Tobias, 1989	L, P	NE, S	AFR, ORI	4, 6, 8	
19	*Asplanchnopushyalinus* Harring, 1913	P	NE	AFR, AUS, NEO, ORI, PAL	4, 18	
20	*Asplanchnopusmulticeps* (Schrank, 1793)	CA, P, RF	NE	AFR, AUS, NEA, NEO, ORI, PAL	4, 39	Incl.: *Asplachnopusmulticeps* (Schrank, 1793) (Ref.4)
21	*Harringiarousseleti* Beauchamp, 1911	PS	S	NEA, NEO, ORI, PAL	24	
	**Family Atrochidae**					
22	*Acyclusinquietus* Leidy, 1882	L	S	NEA, ORI, PAL	30	
23	*Cupelopagisvorax* (Leidy, 1857)	L	C, S	AFR, AUS, NEA, NEO, ORI, PAL	2, 6, 8	
	**Family Brachionidae**					
24	*Anuraeopsiscoelata* Beauchamp, 1932	CA, L, P, PS, RE, RI, SW, TP	N, NE, C, S	AFR, AUS, NEA, NEO, ORI, PAL	4, 5, 6, 8, 18, 19, 21, 22, 23, 24, 25, 31, 36, 41	
25	*Anuraeopsisfissa* (Gosse, 1851)	CA, FF, L, P, PS, RE, RF, RI, SW, TP	N, NE, C, S	AFR, AUS, NEA, NEO, ORI, PAL	1, 2, 3, 4, 5, 6, 8, 10, 11, 13, 14, 16, 18, 21, 22, 23, 24, 25, 27, 31, 36, 39, 40, 41	
26	*Anuraeopsisnavicula* Rousselet, 1911	L, P, PS, RE, RF, TP	NE, C, S	AUS, NEA, NEO, ORI, PAL	4, 6, 8, 11, 22, 24, 25, 27	
27	*Brachionusangularis* Gosse, 1851	CA, FF, L, M, P, PS, RE, RF, RI, SW, TP	N, NE, C, E, S	AFR, AUS, NEA, NEO, ORI, PAL	1, 3, 4, 5, 6, 8, 10, 11, 14, 18, 19, 21, 22, 23, 24, 25, 27, 31, 33, 36, 39, 40, 41	Incl.: BrachionusangularisGossef.typica, f. chelonis (Ref.4)
28	*Brachionusangularisbidens* Plate, 1886	L, M, P, RE, RI, SW	N, NE, C, S	AFR, AUS, NEA, ORI, PAL	4, 31, 33	Incl.: Brachionusangularisf.bidens (Ref.4,31)
29	*Brachionusbennini* Leissling, 1924	P, RI	N, NE	AFR, AUS, NEA, ORI, PAL	4, 31	
30	*Brachionusbidentatus* Anderson, 1889	CA, L, P, RE, RI, SW, TP	N, NE, C	AFR, ANT, AUS, NEA, NEO, ORI, PAL	4, 14, 18, 22, 25, 31	Incl.: Brachionusbidentatusf.inermis (Ref.31)
31	*Brachionusbudapestinensis* Daday, 1885	P, RE, RF, TP	NE, C	AFR, AUS, NEA, NEO, ORI, PAL	4, 25, 31, 40, 41	
32	*Brachionuscalyciflorus* Pallas, 1766	CA, FF, IT, L, M, P, PS, RE, RF, RI, SW, TP	N, NE, C, E, W, S	AFR, ANT, AUS, NEA, NEO, ORI, PAL	1, 3, 4, 6, 8, 10, 14, 18, 19, 21, 22, 23, 24, 25, 27, 31, 33, 36, 40, 41	Incl.: Brachionuscalyciflorusf.typica, f. monstruosus, f. amphiceros (Ref.4); f. anuraeiformis (Ref.31)
33	*Brachionuscaudatus* Barrois & Daday, 1894	CA, FF, L, M, P, PS, RE, RF, RI, SW, TP	N, NE, C, E, S	AFR, AUS, NEA, NEO, ORI, PAL	1, 3, 4, 6, 8, 10, 18, 21, 22, 23, 25, 26, 27, 31, 33, 36, 40, 41	Incl.: Brachionuscaudatusf.aculeatus (Ref.4,23,31), Brachionuscaudatusf.apsteini (Ref.23,26,31), Brachionuscaudatusf.personatus (Ref.4)
34	*Brachionusdichotomus* Shephard, 1911	L, PS, RE	NE, C, S	AUS, ORI	6, 8, 22, 24	
35	*Brachionusdichotomusreductus* Koste & Shiel, 1980	CA, L, M, P, RE, RF, SW, TP	N, NE, C, E, S	AUS, ORI	4, 5, 10, 14, 18, 19, 21, 23, 25, 31, 33	Incl.: BrachionusdichotomusShephardf.reductus Koste & Shiel, 1980 (Ref.4,5,10,14,18,21,23,25,31)
36	*Brachionusdiversicornis* (Daday, 1883)	CA, L, P, RE, RF, RI, SW, TP	N, NE, C, E	AFR, AUS, NEO, ORI, PAL	3, 4, 10, 18, 21, 22, 23, 25, 27, 31, 33, 36	
37	*Brachionusdonneri* Brehm, 1951	CA, IT, L, P, PS, RE, RI, SW, TP	N, NE, C, S	ORI (Known from Thailand, Cambodia and India)	3, 4, 5, 6, 8, 10, 14, 18, 21, 23, 24, 25, 31, 33, 36, 41	
38	*Brachionusdurgae* Dhanapathi, 1974	L, P, TP	NE, C	AFR, NEO, ORI, PAL	4, 25, 31	
39	*Brachionusfalcatus* Zacharias, 1898	CA, IT, L, M, P, PS, RE, RF, RI, SW, TP	N, NE, C, E, S	AFR, AUS, NEA, NEO, ORI, PAL	1, 3, 4, 5, 6, 8, 10, 11, 14, 18, 19, 21, 22, 23, 24, 25, 27, 29, 31, 33, 36, 40, 41	
40	*Brachionusforficula* Wierzejski, 1891	CA, FF, L, M, P, PS, RE, RF, RI, SW, TP	N, NE, C, E, W, S	AFR, AUS, ORI, PAL	1, 3, 4, 6, 8, 10, 14, 18, 19, 21, 22, 23, 24, 25, 27, 29, 31, 33, 36, 41	Incl.: Brachionusforficulaf.typica , f. reductus (Ref.4), Brachionusforficulaf.minor (Ref.31)
41	*Brachionuskostei* Shiel, 1983	CA, L, M, PS, RI	N, NE, S	AUS, ORI, PAL	4, 10, 18, 33	
42	*Brachionusleydigii* Cohn, 1862	FF	C	AFR, AUS, ORI, PAL	3	
43	*Brachionuslyratus* Shephard, 1911	CA, P, PS, RF, TP	NE, C, S	AUS, ORI	4, 18, 24, 25, 40, 41	
44	*Brachionusmurphyi* Sudzuki, 1989	CA, L, P, PS, RF	N, NE, S	ORI (Known from Singapore, Thailand, China and India)	4, 6, 8, 18, 24, 31	Syn.: *Brachionusniwati* Sanoamuang, Segers & Dumont, 1995 (Ref.4,6,8)
45	*Brachionusnilsoni* Ahlstrom, 1940	L, RI, SW	N, C	AFR, AUS, NEA, NEO, ORI, PAL	23, 31	
46	*Brachionusplicatilis* Müller, 1786	FF, L, P, RE	NE	AFR, AUS, NEA, NEO, ORI, PAC, PAL	3, 4	
47	*Brachionusquadridentatus* Hermann, 1783	CA, FF, L, M, P, PS, RE, RF, RI, SW, TP	N, NE, C, E, S	AFR, ANT, AUS, NEA, NEO, ORI, PAC, PAL	1, 3, 4, 6, 8, 10, 11, 13, 14, 16, 18, 19, 21, 22, 23, 24, 25, 27, 31, 33, 36, 39, 40, 41	Incl.: Brachionusquadridentatusf.typica (Ref.4), Brachionusquadridentatusf.brevispinus (Ref.4,19,31)
48	*Brachionusrotundiformis* Tschugunoff, 1921	CA, M, P, PS, RF, RI	NE, C, E, S	AFR, NEA, ORI, PAL	19, 20, 24, 27, 31, 33	
49	*Brachionusrubens* Ehrenberg, 1838	CA, L, P, PS, RE, RF, RI, TP	N, NE, C, S	AFR, NEA, NEO, ORI, PAL	4, 6, 8, 11, 18, 19, 21, 25, 27, 31	
50	*Brachionussericus* Rousselet, 1907	no information	no information	AFR, AUS, ORI, PAL	19	
51	*Brachionussessilis* Varga, 1951	CA, P	NE	AFR, AUS, NEO, ORI, PAL	4	
52	*Brachionussrisumonae* Segers, Kothetip & Sanoamuang, 2004	CA, D, P, SW	NE	ORI (Known from Thailand and India)	18	
53	*Brachionusurceolaris* Müller, 1773	CA, FF, L, PS, RF	NE, C, S	AFR, ANT, AUS, NEA, NEO, ORI, PAL	3, 4, 15, 19, 20, 24, 27, 29, 31	
54	*Brachionusvariabilis* Hempel, 1896	PS	S	AUS, NEA, NEO, ORI, PAL	11	
55	*Keratellacochlearis* (Gosse, 1851)	CA, IT, L, M, P, PS, RE, RF, RI, SW, TP	N, NE, C, E, S	AFR, ANT, AUS, NEA, NEO, ORI, PAL	1, 3, 4, 5, 6, 8, 10, 14, 18, 19, 21, 22, 23, 24, 25, 31, 33, 36, 39, 40, 41	Incl.: Keratellacochlearisf.typica , f. micracantha (Ref.4)
56	*Keratellaedmondsoni* Ahlstrom, 1943	CA, L, P, RE, SW	NE	ORI (Known from Thailand, Cambodia and India)	4, 14, 18, 19, 31	
57	*Keratellajavana* Hauer, 1937	PS	S	AFR, AUS, ORI, PAL	13, 16	
58	*Keratellalenzi* Hauer, 1953	CA, L, P, PS, RE, RF, RI, SW, TP	N, NE, C, S	AFR, NEA, NEO, ORI	2, 4, 6, 8, 10, 14, 18, 19, 21, 22, 23, 25, 27, 31, 33, 36, 39	
59	*Keratellamixta* (Oparina-Charitonova, 1924)	PS	S	NEA, ORI, PAL	16	
60	*Keratellaprocurva* (Thorpe, 1891)	CA, FF, L, P, SW	N, NE, C	AFR, AUS, NEO, ORI, PAL	3, 4, 5, 18, 19, 21, 23, 31	
61	*Keratellataksinensis* Chittapun, Pholpunthin & Segers, 2002	PS	S	ORI (Endemic to Thailand)	16	
62	*Keratellatecta* (Gosse, 1851)	CA, L, M, P, RE, RF, RI, SW	N, NE, C, E	AFR, AUS, NEA, NEO, ORI, PAL	4, 18, 19, 21, 22, 23, 31, 33, 39	
63	*Keratellatropica* (Apstein, 1907)	CA, IT, L, M, P, PS, RE, RF, RI, SW, TP	N, NE, C, E, W, S	AFR, AUS, NEA, NEO, ORI, PAL	1, 2, 3, 4, 5, 6, 8, 10, 11, 14, 18, 19, 21, 22, 23, 24, 25, 27, 31, 33, 36, 39, 40, 41	Syn.: *Keratellavalgatropica* Apstein, 1907 (Ref.2), *Kellatellavalga* (Ehrenberg, 1834) (Ref.3)
64	*Plationuspatulus* (Müller, 1786)	CA, L, M, P, PS, RE, RF, RI, SW, TP	N, NE, C, E, S	AFR, AUS, NEA, NEO, ORI, PAL	3, 4, 6, 8, 10, 11, 13, 14, 16, 18, 19, 21, 22, 23, 24, 25, 27, 31, 33, 36, 39, 40, 41	Syn.: *Brachionuspatulus* Müller, 1786 (Ref.3), Plationuspatulus(Müller)f.typica (Ref.4)
65	*Platyiasleloupi* Gillard, 1957*	P, RF, SW	N, NE, S	AFR, AUS, NEA, NEO, ORI	33, 39	
66	*Platyiasquadricornis* (Ehrenberg, 1832)	CA, FF, L, M, P, PS, RE, RF, RI, SW, TP	N, NE, C, E, S	AFR, AUS, NEA, NEO, ORI, PAC, PAL	3, 4, 6, 8, 10, 11, 14, 16, 18, 19, 22, 23, 24, 25, 27, 31, 33, 39, 40, 41	Incl.: Platyiasquadricornisf.brevispinus (Ref.16)
	**Family Collothecidae**					
67	*Collothecaalgicola* (Hudson, 1886)	L	S	ORI, PAL	30, 32	
68	*Collothecaambigua* (Hudson, 1883)	L	S	AFR, AUS, NEA, NEO, ORI, PAL	30, 32	
69	*Collothecacampanulata* (Dobie, 1849)	CA, L, RE	NE, S	AFR, ANT, AUS, NEA, NEO, ORI, PAL	4, 30, 32	Incl.: Collothecacampanulatavar.longicaudata (Ref.30)
70	*Collothecaedentata* (Collins, 1872)	no information	C	AUS, NEA, NEO, ORI, PAL	2	
71	*Collothecaferox* (Penard, 1914)*	no information	no information	ORI, PAC, PAL	32	
72	*Collothecaheptabrachiata* (Schoch, 1869)	L	S	AFR, NEA, NEO, ORI, PAL	30, 32	
73	*Collothecaorchidacea* Meksuwan, Pholpunthin & Segers, 2013*	L	S	ORI (Endemic to Thailand)	32	
74	*Collothecaornata* (Ehrenberg, 1830)	L, SW	S	AFR, ANT, AUS, NEA, NEO, ORI, PAC, PAL	30, 32	
75	*Collothecastephanochaeta* Edmondson, 1936	L	S	NEA, ORI, PAL	30, 32	
76	*Collothecatenuilobata* (Anderson, 1889)	L	NE, S	AFR, AUS, NEA, NEO, ORI, PAL	14, 30, 32	
77	*Collothecatrilobata* (Collins, 1872)	L, SW	C, S	AFR, AUS, NEA, ORI, PAL	23, 30, 32	Incl.: Collothecacf.trilobata (Collins, 1872) (Ref.23)
78	*Stephanocerosfimbriatus* (Goldfusz, 1820)	L	S	AUS, NEA, NEO, ORI, PAL	30, 32	
79	*Stephanocerosmillsii* (Kellicott, 1885)	L	S	NEA, ORI	30, 32	Incl.: Stephanocerosfimbriatusvar.millsii (Kellicott, 1885) (Ref.30)
	**Family Conochilidae**					
80	*Conochiluscoenobasis* (Skorikov, 1914)	L, P, RE, TP	NE	AFR, AUS, NEA, NEO, ORI, PAL	4, 14, 21, 25	
81	*Conochilusdossuarius* Hudson, 1885	CA, P, RF	N, NE	AFR, AUS, NEA, NEO, ORI, PAL	4, 10	
82	*Conochilushippocrepis* (Schrank, 1803)	L, P, TP	NE, S	AFR, AUS, NEA, NEO, ORI, PAL	4, 21, 25, 30	
83	*Conochilusnatans* (Seligo, 1900)	CA, L, PS, TP	NE, S	AFR, AUS, NEA, NEO, ORI, PAL	4, 14, 21, 24, 25	
84	*Conochilusunicornis* Rousselet, 1892	L, RE	NE, C	AFR, AUS, NEA, NEO, ORI, PAL	3, 14	
	**Family Dicranophoridae**					
85	*Aspeltacircinator* (Gosse, 1886)	L	NE	AUS, NEA, NEO, ORI, PAL	14	
86	*Dicranophoroidescaudatus* (Ehrenberg, 1834)	CA, L, P	N, NE	AFR, AUS, NEA, NEO, ORI, PAL	4, 10, 14, 18, 21	Syn.: *Dicranophoruscaudatus* (Ehrenberg, 1834) (Ref.4,14)
87	*Dicranophoroidesclaviger* (Hauer, 1965)	L, P, TP	NE, S	AFR, NEO, ORI	4, 6, 8, 14, 18, 25	Syn.: *Dicranophorusclaviger* (Hauer) (Ref.4,6,8)
88	*Dicranophorusepicharis* Harring & Myers, 1928	L, P, PS, RF	NE, C, S	AFR, AUS, NEA, NEO, ORI, PAL	2, 4, 11, 13, 14, 18, 21, 24, 27	Incl.: Dicranophoruscf.epicharis Harring & Myers, 1928 (Ref.4)
89	*Dicranophorusgrandis* (Ehrenberg, 1832)	CA, L, P	N, NE	AFR, AUS, NEA, NEO, ORI, PAC, PAL	4, 10, 14	
90	*Dicranophorusprionacis* Harring & Myers, 1928	L	S	AFR, NEA, NEO, ORI, PAL	6, 8	
91	*Dicranophorusrobustus* Harring & Myers, 1928	FF	C	AFR, AUS, NEA, NEO, ORI, PAL	3	
92	*Encentrumlongidens* Donner, 1943	PS	S	ORI, PAL	15	
93	*Encentrumpornsilpi* Segers & Chittapun, 2001	PS	S	ORI (Endemic to Thailand)	15, 20, 24	
	**Family Epiphanidae**					
94	*Cyrtoniatuba* (Ehrenberg, 1834)	L	S	AFR, AUS, NEA, NEO, ORI, PAC, PAL	6, 8	
95	*Epiphanesclavulata* (Ehrenberg, 1831)	CA, L, P, RF, TP	N, NE	AFR, AUS, NEA, NEO, ORI, PAL	4, 10, 18, 21, 25	
96	*Epiphanesmacroura* (Barrois & Daday, 1894)	CA, L, P	NE	AFR, AUS, NEA, NEO, ORI, PAL	4	Incl.: *Epiphanesmacrourus* (Barrois & Daday, 1894) (Ref.4)
97	*Proalidessubtilis* Rodewald, 1940	P	NE	AFR, AUS, NEO, ORI, PAL	4	
98	*Proalidestentaculatus* Beauchamp, 1907	P	NE	AFR, NEA, NEO, ORI, PAC, PAL	4	
	**Family Euchlanidae**					
99	*Beauchampiellaeudactylota* (Gosse, 1886)	CA, P, PS, RF, RI	N, NE, S	AFR, AUS, NEA, NEO, ORI, PAL	4, 10, 16, 18, 39	Syn.: *Manfrediumeudactylotum* (Gosse, 1886) (Ref.4,10,16,18)
100	*Dipleuchlanispropatula* (Gosse, 1886)	CA, L, P, PS, RE, RF, RI, TP	N, NE, C, S	AFR, AUS, NEA, NEO, ORI, PAL	3, 4, 6, 8, 10, 13, 14, 16, 18, 21, 24, 25, 27, 39, 40	Incl.: Dipleuchlanispropatulaf.macrodactyla (Ref.16)
101	*Euchlanisdilatata* Ehrenberg, 1832	CA, FF, L, P, PS, RE, RF, RI, SW, TP	N, NE, C, S	AFR, ANT, AUS, NEA, NEO, ORI, PAC, PAL	1, 2, 3, 4, 6, 8, 10, 11, 13, 14, 18, 21, 23, 24, 25, 27, 40	
102	*Euchlanisincisa* Carlin, 1939	L, P, PS, RF, SW, TP	N, NE, C, S	AFR, AUS, NEA, NEO, ORI, PAC, PAL	4, 10, 11, 13, 14, 16, 18, 21, 23, 25, 39	Incl.: Euchlanisincisaf.mucronata (Ref.11)
103	*Euchlanislyra* Hudson, 1886	PS	S	AFR, AUS, NEA, NEO, ORI	11	Incl.: Euchlanislyraf.myersi (Ref.11)
104	*Euchlanismeneta* Myers, 1930	L, PS	NE, C, S	AFR, AUS, NEA, NEO, ORI, PAL	2, 11, 14	
105	*Euchlanistriquetra* Ehrenberg, 1838	no information	C	AFR, AUS, NEA, NEO, ORI, PAL	2	
106	*Tripleuchlanisplicata* (Levander, 1894)	CA, FF, L, P, RE, RF, SW	N, NE, C	AFR, AUS, NEA, NEO, ORI, PAC, PAL	1, 3, 4, 10, 14, 18, 22, 23, 39	
	**Family Flosculariidae**					
107	*Beauchampiacrucigera* (Dutrochet, 1812)	L	C, S	AFR, AUS, NEA, NEO, ORI, PAC, PAL	2, 30	Syn.: *Beauchampiacrucigeracrucigera* (Dutrochet, 1812) (Ref.2)
108	*Flosculariaarmata* Segers, 1997	L	S	NEA, NEO, ORI	30	
109	*Flosculariabifida* Segers, 1997	L	S	NEA, NEO, ORI	30	
110	*Flosculariaconifera* (Hudson, 1886)	L, PS	NE, C, S	AFR, AUS, NEA, NEO, ORI, PAL	2, 13, 14, 18, 20, 24, 30	
111	*Flosculariadecora* Edmondson, 1940	no information	C	NEO, ORI	2	
112	*Flosculariamelicerta* (Ehrenberg, 1832)	no information	C	AFR, AUS, NEA, NEO, ORI, PAL	2	
113	*Flosculariapedunculata* (Joliet, 1883)	L	S	NEA, ORI, PAL	30	
114	*Flosculariaringens* (Linnaeus, 1758)	L	NE, C, S	AFR, AUS, NEA, NEO, ORI, PAL	2, 14, 30	
115	*Flosculariawallacei* Segers & Shiel, 2008	L	S	AUS, ORI	30	
116	*Lacinulariaflosculosa* (Müller, 1773)	L	S	AUS, NEA, NEO, ORI, PAL	30	
117	*Lacinularoidescoloniensis* (Colledge, 1918)	L	S	AUS, NEA, NEO, ORI, PAL	30	
118	*Limniasceratophylli* Schrank, 1803	L	C, S	AFR, AUS, NEA, NEO, ORI, PAC, PAL	2, 30	It is a species complex.
119	*Limniaslenis* Meksuwan, Jaturapruek & Maiphae, 2018*	PS	S	ORI (Endemic to Thailand)	35	
120	*Limniasmelicerta* Weisse, 1848	L, PS, SW	NE, C, S	AFR, AUS, NEA, NEO, ORI, PAC, PAL	2, 15, 30, 35	It is a species complex.
121	*Limniasnovemceras* Meksuwan, Jaturapruek & Maiphae, 2018*	RI	S	ORI (Endemic to Thailand)	35	
122	*Octotrochaspeciosa* Thorpe, 1893	L	S	AUS, NEA, NEO, ORI, PAL	30	
123	*Pentatrochagigantea* Segers & Shiel, 2008	L	S	AUS, ORI	30	
124	*Ptyguraagassizi* Edmondson, 1948	L	S	NEA, ORI	30	
125	*Ptygurabarbata* Edmondson, 1939	L	S	AUS, NEA, NEO, ORI	30	
126	*Ptygurabeauchampi* Edmondson, 1940	L	S	NEA, NEO, ORI, PAL	30	
127	*Ptygurabrachiata* (Hudson, 1886)	no information	C	AFR, AUS, NEA, NEO, ORI, PAL	2	
128	*Ptyguracrystallina* (Ehrenberg, 1834)	L	S	AFR, ANT, AUS, NEA, NEO, ORI, PAL	30	
129	*Ptyguractenoida* Koste & Tobias, 1990	L	S	AFR, AUS, ORI, PAL	30	
130	*Ptyguraelsteri* Koste, 1972	L	C, S	NEO, ORI	2, 30	Syn.: *Ptyguraelsterielsteri* Koste, 1972 (Ref.2)
131	*Ptygurafurcillata* (Kellicott, 1889)	L	NE, C, S	AFR, AUS, NEA, NEO, ORI, PAL	2, 14, 30	Syn.: *Ptygurafurcillatafurcillata* (Kellicott, 1889) (Ref.2), Incl.: Ptygurafurcillataf.variabilis (Ref.2)
132	*Ptygurakostei* José de Paggi, 1996	L	NE	AFR, NEO, ORI	2, 14	Syn.: Ptyguraelsterif.thailandensis Koste, 1975 (Ref.2,14)
133	*Ptyguralongicornis* (Davis, 1867)	L	S	AUS, NEA, NEO, ORI, PAL	30	
134	*Ptyguramelicerta* Ehrenberg, 1832	L	NE, C	AFR, ANT, AUS, NEA, NEO, ORI, PAL	2, 14	
135	*Ptyguramucicola* (Kellicott, 1888)	L	C, S	AFR, AUS, NEA, NEO, ORI, PAL	2, 30	
136	*Ptyguranoodti* (Koste, 1972)	L	S	NEO, ORI	30	
137	*Ptygurapedunculata* Edmondson, 1939	L	S	NEA, NEO, ORI, PAL	30	
138	*Ptyguratacita* Edmondson, 1940	L	NE, S	AUS, NEA, NEO, ORI	14, 30	
139	*Ptygurathalenoiensis* Meksuwan, Pholpunthin & Segers, 2011	L	S	ORI (Known from Thailand and Cambodia)	30	
140	*Ptygurawilsonii* (Anderson & Shephard, 1892)	L	S	AFR, AUS, NEO, ORI, PAL	30	
141	*Sinantherinaariprepes* Edmondson, 1939	L, SW	NE, C	AUS, NEA, NEO, ORI	14, 21, 23	Incl.: *Sinantherinaariprepes* Edmondson, 1939 (Ref.14,21), *S.areprepes* Edmondson, 1939 (Ref.23)
142	*Sinantherinasemibullata* (Thorpe, 1889)	CA, L, P, RF	N, NE, S	AFR, AUS, NEA, NEO, ORI, PAL	4, 10, 21, 30	
143	*Sinantherinasocialis* (Linnaeus, 1758)	L	C, S	AFR, AUS, NEA, NEO, ORI, PAL	2, 30	
144	*Sinantherinaspinosa* (Thorpe, 1893)	L, P, RF, SW, TP	NE, C, S	AFR, AUS, NEA, NEO, ORI, PAL	4, 14, 18, 21, 23, 25, 27, 30, 39	
	**Family Gastropodidae**					
145	*Ascomorphaagilis* Zacharias, 1893	RE	C	AFR, ORI, PAL	22	
146	*Ascomorphaecaudis* Perty, 1850	CA, L, P, RE, RI	N, NE, C, S	AFR, AUS, NEA, NEO, ORI, PAL	4, 6, 8, 10, 21, 22	
147	*Ascomorphaovalis* (Bergendal, 1892)	CA, L, P, PS, RE, RI, SW, TP	NE, C, S	AFR, AUS, NEA, NEO, ORI, PAL	3, 4, 8, 14, 18, 21, 22, 23, 24, 25	
148	*Ascomorphasaltans* Bartsch, 1870	FF, L, P, RE, SW	NE, C, S	AFR, AUS, NEA, NEO, ORI, PAL	3, 4, 6, 8, 14, 18, 21, 22, 23	
149	*Gastropushyptopus* (Ehrenberg, 1838)	L, P, RI	NE	AFR, AUS, NEA, NEO, ORI, PAL	4	
	**Family Hexarthridae**					
150	*Hexarthrafennica* (Levander, 1892)	L, P	NE	AFR, AUS, NEA, NEO, ORI, PAL	4	
151	*Hexarthraintermedia* (Wiszniewski, 1929)	CA, L, P, RE, RI, SW, TP	N, NE, C, S	AFR, AUS, NEA, NEO, ORI, PAL	4, 5, 6, 8, 10, 14, 18, 21, 22, 23, 25	
152	*Hexarthramira* (Hudson, 1871)	CA, L, P, PS, RF, RI, SW, TP	N, NE, C, S	AFR, AUS, NEA, NEO, ORI, PAC, PAL	4, 6, 8, 10, 14, 18, 20, 21, 23, 24, 25, 39	
153	*Hexarthraoxyuris* (Zernov, 1903)	P	NE	AFR, AUS, NEA, NEO, ORI, PAC, PAL	4	
	**Family Ituridae**					
154	*Ituraaurita* (Ehrenberg, 1830)	P	NE	AFR, AUS, NEA, NEO, ORI, PAL	4	
155	*Iturasymmetrica* Segers, Mbogo & Dumont, 1994	L	NE	AFR, AUS, ORI	4	
	**Family Lecanidae**					
156	*Lecaneabanica* Segers, 1994	P, PS	S	AFR, ORI, PAL	13, 24, 28, 33	
157	*Lecaneacanthinula* (Hauer, 1938)	P, PS (they have been recorded in slightly brackish water)	NE, S	ORI, PAL	4, 7, 15, 24, 28	
158	*Lecaneaculeata* (Jakubski, 1912)	CA, FF, L, P, PS, RE, RF, SW, TP	N, NE, C, S	AFR, AUS, NEA, NEO, ORI, PAC, PAL	2, 3, 4, 6, 8, 9, 10, 11, 13, 14, 16, 18, 21, 22, 23, 24, 25, 28, 29, 33, 40	
159	*Lecaneacus* (Harring, 1913)	FF, P, SW	C, S	AUS, NEA, NEO, ORI, PAL	1, 3, 33	
160	*Lecaneaeganea* Harring, 1914	FF, P, RE, RF	N, NE, C	AFR, AUS, NEA, NEO, ORI	3, 4, 10, 22, 28	Incl.: *Lecaneaegana* Harring, 1914 (Ref.22)
161	*Lecanearcuata* (Bryce, 1891)	FF, P, PS	NE, C, S	AFR, ANT, AUS, NEA, NEO, ORI, PAC, PAL	1, 2, 3, 4, 11, 13, 28	
162	*Lecanearcula* Harring, 1914	L, P, PS, RE, RF, SW, TP	N, NE, C, S	AFR, AUS, NEA, NEO, ORI, PAL	4, 6, 8, 9, 10, 11, 14, 16, 18, 21, 22, 23, 24, 25, 28, 33, 39	Syn.: *Lecanestrandi* Bērziņš, 1943 (Ref.22)
163	*Lecanearmata* Thomasson, 1971	RE	E	NEO, ORI	28	
164	*Lecaneaspasia* Myers, 1917	CA, L, P, RF	N, NE	NEA, NEO, ORI, PAL	4, 10, 18, 28	
165	*Lecanebaimaii* Sanoamuang & Savatenalinton, 1999	CA, RE, RF, SW	NE	AFR, ORI	12, 18, 28, 39	
166	*Lecanebatillifer* (Murray, 1913)	L, P, PS, RE, SW	NE, C, S	AUS, ORI	4, 6, 8, 18, 21, 23, 24, 28, 33	
167	*Lecanebifastigata* Hauer, 1938	P, SW	NE, C	ORI, PAL	4, 7, 23, 28	
168	*Lecanebifurca* (Bryce, 1892)	L, PS, TP	NE, S	AFR, AUS, NEA, NEO, ORI, PAC, PAL	6, 8, 11, 20, 21, 24, 25, 28	
169	*Lecaneblachei* Bērziņš, 1973	CA, D, L, P, RE	N, NE, C, S	ORI (Known from Cambodia, Indonesian Borneo, India and Thailand)	4, 7, 9, 10, 14, 18, 21, 28	
170	*Lecanebraumi* Koste, 1988	L, PS, SW	NE, E, S	AFR, AUS, ORI	5, 13, 14, 16, 28, 33	
171	*Lecanebulla* (Gosse, 1851)	CA, D, FF, L, M, P, PS, RE, RF, RI, SW, TP	N, NE, C, E, S	AFR, AUS, NEA, NEO, ORI, PAC, PAL	1, 2, 3, 4, 5, 6, 8, 9, 10, 11, 13, 14, 15, 16, 18, 20, 21, 22, 23, 24, 25, 27, 28, 29, 33, 36, 39, 40, 41	Syn.: *Lecanebullabulla* (Gosse, 1851) (Ref.28)
172	*Lecanebulladiabolica* (Hauer, 1936)	L	S	ORI (Known from Thailand and India)	28	
173	*Lecanecalcaria* Harring & Myers, 1926	L, PS	NE, S	NEA, ORI	8, 13, 18, 28	
174	*Lecanechinesensis* Zhuge & Koste, 1996	RE, RF	C, S	ORI, PAL	28, 40	
175	*Lecaneclara* (Bryce, 1892)	L, M, P, PS	NE, S	AFR, NEA, NEO, ORI, PAC, PAL	6, 8, 13, 21, 28, 33	
176	*Lecaneclosterocerca* (Schmarda, 1859)	CA, FF, L, P, PS, RE, RF, SW, TP	N, NE, C, S	AFR, ANT, AUS, NEA, NEO, ORI, PAC, PAL	1, 2, 3, 4, 6, 8, 10, 11, 13, 14, 16, 18, 21, 22, 23, 24, 25, 27, 28, 33, 39, 40	
177	*Lecanecornuta* (Müller, 1786)	FF, P	C	NEA, NEO, ORI, PAL	1, 2, 3, 41	
178	*Lecanecrenata* (Harring, 1913)	L, PS, SW	NE, C, E, S	AFR, AUS, NEA, NEO, ORI, PAL	2, 28, 33	
179	*Lecanecrepida* Harring, 1914	CA, L, P, PS, RE, RF, SW, TP	N, NE, C, S	AFR, AUS, NEA, NEO, ORI, PAL	2, 4, 6, 8, 10, 11, 13, 14, 18, 21, 23, 24, 25, 28, 33, 39	
180	*Lecanecurvicornis* (Murray, 1913)	CA, D, FF, L, M, P, PS, RE, RF, RI, SW, TP	N, NE, C, E, S	AFR, AUS, NEA, NEO, ORI, PAL	1, 3, 4, 6, 8, 9, 10, 13, 14, 16, 18, 21, 22, 23, 24, 25, 27, 28, 29, 33, 36, 39, 40, 41	Incl.: Lecanecurvicornisf.typica (Ref.4)
181	*Lecanedecipiens* (Murray, 1913)	CA, L, P, PS, RE, RI, SW, TP	NE, S	AFR, AUS, NEA, NEO, ORI, PAC, PAL	11, 12, 13, 14, 18, 21, 25, 28, 33	
182	*Lecanedonneri* Chengalath & Mulamoottil, 1974	P, TP	NE	AFR, NEA, ORI	12, 18, 25, 28	
183	*Lecanedoryssa* Harring, 1914	CA, L, P, PS, RF, TP	N, NE, S	AFR, AUS, NEO, ORI, PAL	4, 10, 13, 14, 16, 18, 21, 24, 25, 28	Incl.: Lecanecf.doryssa Harring, 1914 (Ref.14)
184	*Lecaneelegans* Harring, 1914	CA, L, RF, TP	N, NE, C	AFR, NEO, ORI, PAC, PAL	2, 10, 14, 18, 21, 25, 27, 28, 29, 39	
185	*Lecaneenowi* Segers and Mertens, 1997	PS	S	AFR, ORI	16, 28	
186	*Lecaneeswari* Dhanapathi, 1976	P	NE	AFR, ORI	7, 18, 28	
187	*Lecaneflexilis* (Gosse, 1886)	L, P, PS, SW	NE, C, S	AFR, AUS, NEA, NEO, ORI, PAC, PAL	4, 11, 14, 18, 21, 23, 24, 28	
188	*Lecanefurcata* (Murray, 1913)	CA, L, P, PS, RE, RF, RI, SW, TP	N, NE, C, S	AFR, AUS, NEA, NEO, ORI, PAL	2, 4, 6, 8, 9, 10, 11, 13, 14, 16, 18, 21, 22, 23, 24, 25, 28	
189	*Lecanegaleata* (Bryce, 1892)	no information	C	NEA, ORI, PAL	2	Syn.: Lecane (Monostyla) pygmaea Daday, 1897 (Ref.2)
190	*Lecanegrandis* (Murray, 1913)	PS	NE, S	AUS, NEA, NEO, ORI, PAL	11, 12, 24, 28, 33	
191	*Lecanehaliclysta* Harring & Myers, 1926	CA, L, P, PS, RF, SW, TP	N, NE, C, S	AFR, AUS, NEA, NEO, ORI, PAL	4, 10, 13, 14, 18, 21, 23, 24, 25, 28, 33, 39	
192	*Lecanehamata* (Stokes, 1896)	CA, D, L, M, P, PS, RE, RF, RI, SW, TP	N, NE, C, S	AFR, AUS, NEA, NEO, ORI, PAC, PAL	2, 4, 5, 6, 8, 9, 10, 11, 13, 14, 15, 16, 18, 21, 22, 23, 24, 25, 27, 28, 33, 36, 39, 40, 41	
193	*Lecanehastata* (Murray, 1913)	CA, FF, L, P, PS, RE, RF, RI, SW, TP	N, NE, C, E, S	AFR, AUS, NEA, NEO, ORI, PAC, PAL	3, 4, 5, 10, 18, 21, 22, 23, 24, 25, 28, 33, 39	
194	*Lecanehornemanni* (Ehrenberg, 1834)	CA, D, L, P, PS, RE, RF, SW, TP	N, NE, C, S	AFR, AUS, NEA, NEO, ORI, PAC, PAL	4, 5, 6, 8, 9, 10, 13, 14, 16, 18, 21, 22, 23, 24, 25, 27, 28, 33, 39, 40	
195	*Lecaneinermis* (Bryce, 1892)	L, P, PS, RF	NE, C, S	AFR, AUS, NEA, NEO, ORI, PAC, PAL	2, 4, 6, 8, 11, 13, 16, 20, 21, 24, 27, 28	
196	*Lecaneinopinata* Harring & Myers, 1926	FF, L, P, PS, RE, RF, SW, TP	N, NE, C, S	AFR, AUS, NEA, NEO, ORI, PAC, PAL	3, 4, 10, 11, 13, 14, 18, 21, 22, 23, 25, 28, 29, 39	
197	*Lecaneintrasinuata* (Olofsson, 1917)	no information	C	NEA, ORI, PAL	2	
198	*Lecaneisanensis* Sanoamuang & Savatenalinton, 2001	L	NE	ORI (Known from Thailand and India)	14, 28	
199	*Lecanejunki* Koste, 1975	L, PS	C, S	ORI (Endemic to Thailand)	2, 7, 17, 28	
200	*Lecanekunthuleensis* Chittapun, Pholpunthin & Segers, 2003	PS	S	ORI (Known from Thailand, Vietnam and China)	17, 28	
201	*Lecanelamellata* (Daday, 1893)	FF	C	NEA, ORI, PAL	3	
202	*Lecanelateralis* Sharma, 1978	CA, D, L, M, P, PS, RE, RF, RI, SW, TP	N, NE, C, E, S	AFR, AUS, ORI	4, 5, 6, 8, 9, 10, 11, 14, 16, 18, 21, 24, 25, 27, 28, 29, 33, 39, 40	
203	*Lecanelatissima* Yamamoto, 1955	L, P, RE, SW	NE, S	AUS, NEA, ORI, PAL	4, 7, 9, 14, 18, 21, 28, 33	Syn.: *Lecanethailandensis* Segers & Sanoamuang, 1994 (Ref.4,7,9,14,18,21)
204	*Lecanelauterborni* Hauer, 1924	FF	C	AFR, NEA, NEO, ORI, PAC, PAL	3, 28	
205	*Lecaneleontina* (Turner, 1892)	CA, D, L, M, P, PS, RE, RF, RI, SW, TP	N, NE, C, E, S	AFR, AUS, NEA, NEO, ORI, PAL	2, 4, 5, 6, 8, 9, 10, 11, 13, 14, 16, 18, 21, 22, 23, 24, 25, 27, 28, 33, 39, 41	Highly variable morphospecies
206	*Lecaneludwigii* (Eckstein, 1883)	CA, L, P, PS, RE, RF, RI, SW, TP	N, NE, C, S	AFR, AUS, NEA, NEO, ORI, PAC, PAL	2, 4, 6, 8, 9, 10, 11, 13, 14, 16, 18, 20, 21, 23, 24, 25, 28, 33, 39, 41	
207	*Lecaneluna* (Müller, 1776)	CA, D, FF, L, M, P, PS, RE, RF, SW, TP	N, NE, C, E, S	AFR, AUS, NEA, NEO, ORI, PAC, PAL	1, 3, 4, 6, 8, 9, 10, 11, 14, 18, 21, 22, 23, 24, 25, 27, 28, 29, 33, 39, 40, 41	
208	*Lecanelunaris* (Ehrenberg, 1832)	CA, D, FF, L, M, P, PS, RE, RF, SW, TP	N, NE, C, E, S	AFR, ANT, AUS, NEA, NEO, ORI, PAC, PAL	2, 3, 4, 6, 8, 9, 10, 11, 13, 14, 16, 18, 21, 22, 23, 24, 25, 28, 33, 36, 39, 41	Highly variable morphospecies
209	*Lecanelungae* Savatenalinton & Segers, 2005	RE	NE	ORI (Endemic to Thailand)	21, 28	
210	*Lecanemartensi* Savatenalinton & Segers, 2008	no information	C	ORI (Endemic to Thailand)	28	
211	*Lecanemicrognatha* Segers & Savatenalinton, 2010	RF, RI	N, NE	ORI (Endemic to Thailand)	28	
212	*Lecaneminuta* Segers, 1994	L, M, P	S	ORI (Known from Borneo, Thailand, India and Vietnam)	6, 7, 8, 28, 33	
213	*Lecanemitis* Harring & Myers, 1926	PS	S	NEA, NEO, ORI	13, 28	
214	*Lecanemonostyla* (Daday, 1897)	FF, L, P, PS, RF, SW, TP	NE, C, S	AFR, AUS, NEA, NEO, ORI, PAC, PAL	3, 4, 11, 13, 16, 18, 23, 24, 25, 28, 33, 39	
215	*Lecanenana* (Murray, 1913)	FF, L, P, RF	NE, C, S	AFR, AUS, NEA, NEO, ORI, PAL	1, 3, 4, 6, 8, 14, 18, 21, 28, 29, 33	
216	*Lecanenelsoni* Segers, 1994	L	NE	AFR, NEO, ORI	14, 28	
217	*Lecanenitida* (Murray, 1913)	L, P, PS, RE, RF, SW	N, NE, E, S	AFR, AUS, NEO, ORI	4, 28, 33, 39	Syn.: Lecanecurvicornis(Murray)f.nitida (Murray) (Ref.4)
218	*Lecaneniwati* Segers, Kothetip & Sanoamuang, 2004	D, L, RI	NE	ORI (Known from Thailand and India)	18, 28	
219	*Lecaneobtusa* (Murray, 1913)	CA, D, L, M, P, PS, RE, RF, SW, TP	N, NE, C, S	AFR, AUS, NEA, NEO, ORI, PAL	4, 6, 8, 9, 10, 11, 13, 14, 15, 16, 18, 20, 21, 23, 24, 25, 28, 33, 39	
220	*Lecaneopias* (Harring & Myers, 1926)	RI	NE	AFR, NEA, ORI, PAL	21, 28	
221	*Lecanepalinacis* Harring & Myers, 1926	PS, RF	C, S	NEA, ORI, PAC	13, 24, 27, 28, 29	
222	*Lecanepapuana* (Murray, 1913)	CA, D, FF, L, M, P, PS, RE, RF, RI, SW, TP	N, NE, C, E, S	AFR, AUS, NEA, NEO, ORI, PAC, PAL	1, 3, 4, 5, 6, 8, 9, 10, 11, 13, 14, 16, 18, 21, 22, 23, 25, 27, 28, 29, 33, 39, 40, 41	
223	*Lecanepaxiana* Hauer, 1940	RE	NE, C	AFR, ORI, PAC, PAL	21, 28	
224	*Lecanepertica* Harring & Myers, 1926	CA, L, PS	N, NE, S	AFR, AUS, NEA, NEO, ORI	6, 8, 10, 13, 14, 16, 18, 28	
225	*Lecanepunctata* (Murray, 1913)	FF, L, P, RE, SW	NE, C, S	AFR, NEA, NEO, ORI, PAL	3, 12, 21, 22, 23, 28, 33	Syn.: *Lecaneharringi* Ahlstrom, 1934 (Ref.3,22)
226	*Lecanepusilla* Harring, 1914	L, PS, RF	N, NE, S	AFR, AUS, NEO, ORI, PAL	4, 10, 13, 14, 18, 21, 28, 39	
227	*Lecanepyriformis* (Daday, 1905)	L, M, P, PS, RF, SW, TP	N, NE, C, S	AFR, AUS, NEA, NEO, ORI, PAC, PAL	2, 4, 11, 13, 14, 15, 16, 18, 21, 23, 24, 25, 27, 28, 33, 39	
228	*Lecanequadridentata* (Ehrenberg, 1830)	CA, D, FF, L, M, P, PS, RE, RF, RI, SW, TP	N, NE, C, S	AFR, AUS, NEA, NEO, ORI, PAC, PAL	1, 2, 3, 4, 6, 8, 9, 10, 11, 13, 14, 18, 23, 24, 25, 27, 28, 33, 39, 40, 41	
229	*Lecanerhenana* Hauer, 1929	L, P, PS, RE, RF, RI, SW, TP	N, NE, C, S	AFR, NEO, ORI, PAL	4, 6, 8, 9, 10, 14, 18, 21, 23, 24, 25, 27, 28, 33	
230	*Lecanerhytida* Harring & Myers, 1926	CA, L, M, PS, RF, SW	N, NE, C, E, S	AFR, AUS, NEA, NEO, ORI	6, 8, 10, 11, 13, 15, 18, 23, 24, 28, 33	
231	*Lecanerobertsonae* Segers, 1993	PS	NE, S	AUS, NEO, ORI	18, 21, 24, 28	
232	*Lecaneruttneri* Hauer, 1938	L, TP	NE	AFR, NEO, ORI	4, 14, 18, 21, 25, 28	
233	*Lecanesegersi* Sanoamuang, 1996	PS, RF, SW	NE, C, S	ORI (Endemic to Thailand)	5, 7, 15, 18, 21, 24, 27, 28	
234	*Lecaneserrata* (Hauer, 1938)	no information	NE	AFR, AUS, ORI	12, 18, 28	
235	*Lecaneshieli* Segers & Sanoamuang, 1994	L, PS, RE, RI	NE, S	AUS, ORI, PAL	4, 7, 11, 14, 18, 21, 28	
236	*Lecanesignifera* (Jennings, 1896)	CA, D, L, M, P, PS, RE, RF, RI, SW, TP	N, NE, C, E, S	AFR, AUS, NEA, NEO, ORI, PAL	2, 4, 5, 6, 8, 9, 10, 11, 13, 14, 16, 18, 21, 22, 23, 24, 25, 27, 28, 33, 39, 40, 41	Incl.: *Lecaneploenensis* (Voigt, 1902) (Ref.2)
237	*Lecanesimonneae* Segers, 1993	L, PS	NE, S	AFR, ORI	13, 14, 16, 18, 28	
238	*Lecanesola* Hauer, 1936	FF, P, TP	NE	NEO, ORI	3, 4, 18, 21, 25, 28	
239	*Lecanestenroosi* (Meissner, 1908)	CA, L, P, PS, RE, RF, RI, SW	N, NE, C, S	AFR, AUS, NEA, NEO, ORI, PAL	2, 4, 9, 10, 11, 18, 21, 22, 23, 27, 28, 33, 39, 40	Incl.: Lecanestenroosif.lineata (Meissner, 1908) (Ref.22)
240	*Lecanestichaea* Harring, 1913	RE	C	AFR, AUS, NEA, NEO, ORI, PAC, PAL	2, 22, 28	
241	*Lecanestichoclysta* Segers, 1993	RE	NE	AFR, ORI	21, 28	
242	*Lecanesubtilis* Harring & Myers, 1926	PS	NE, S	AFR, NEA, NEO, ORI, PAL	18, 24, 28	
243	*Lecanesuperaculeata* Sanoamuang & Segers, 1997	CA, P, PS, RE, SW	N, NE, C, S	ORI (Known from Thailand, India, Vietnam and Cambodia)	7, 10, 16, 18, 24, 28, 33	
244	*Lecanesympoda* Hauer, 1929	L	S	AFR, ORI, PAL	6, 8, 28	
245	*Lecanesyngenes* (Hauer, 1938)	PS	S	AFR, AUS, NEO, ORI, PAC	13, 28	
246	*Lecanetenuiseta* Harring, 1914	L, P, PS, RE, RF, RI, SW, TP	NE, C, S	AFR, ANT, AUS, NEA, NEO, ORI, PAC, PAL	2, 4, 6, 8, 9, 11, 13, 14, 16, 18, 20, 21, 22, 23, 24, 25, 28, 29, 39	
247	*Lecanethalera* (Harring & Myers, 1926)	L, P, SW	NE, C, S	AUS, NEA, NEO, ORI, PAL	4, 23, 28, 33	
248	*Lecanethienemanni* (Hauer, 1938)	P, PS, RE, RF, RI, SW, TP	NE, C, S	AFR, AUS, NEO, ORI	12, 13, 18, 21, 22, 23, 24, 25, 27, 28, 39	
249	*Lecaneundulata* Hauer, 1938	CA, L, P, PS, RF, TP	N, NE, C, S	AFR, AUS, NEA, NEO, ORI, PAL	4, 6, 8, 10, 13, 14, 18, 21, 24, 25, 28, 29, 33	
250	*Lecaneunguitata* (Fadeev, 1925)	CA, D, L, M, P, PS, RE, RF, RI, SW, TP	N, NE, C, E, S	AFR, AUS, ORI, PAL	4, 6, 8, 9, 10, 11, 13, 14, 16, 18, 20, 21, 23, 24, 25, 27, 28, 33, 39, 40	
251	*Lecaneungulata* (Gosse, 1887)	CA, D, L, M, P, PS, RE, RF, RI, SW, TP	N, NE, C, E, S	AFR, AUS, NEA, NEO, ORI, PAL	2, 4, 6, 8, 9, 10, 11, 13, 14, 16, 18, 21, 22, 23, 24, 25, 27, 28, 33, 36, 40, 41	
	**Family Lepadellidae**					
252	*Colurellaadriatica* Ehrenberg, 1831	FF, L, P, PS, TP	NE, C, S	AFR, ANT, AUS, NEA, NEO, ORI, PAC, PAL	1, 3, 4, 13, 14, 21, 24, 25	
253	*Colurellacolurus* (Ehrenberg, 1830)	L, PS, RF	N, NE, C, S	AFR, ANT, AUS, NEA, NEO, ORI, PAL	2, 4, 10, 11, 13, 16, 24, 27	
254	*Colurellacoluruscompressa* (Lucks, 1912)	PS	S	ANT, AUS, NEA, NEO, ORI, PAL	15	
255	*Colurellahindenburgi* Steinecke, 1917	RE	C	AFR, AUS, NEA, NEO, ORI, PAC, PAL	22	
256	*Colurellaobtusa* (Gosse, 1886)	L, P, PS, RE, RF, TP	NE, C, S	AFR, ANT, AUS, NEA, NEO, ORI, PAC, PAL	2, 4, 6, 8, 11, 13, 14, 15, 16, 18, 21, 22, 24, 25, 39	
257	*Colurellapsammophila* Segers & Chittapun, 2001	PS	S	ORI (Known from Thailand and China)	15, 24	
258	*Colurellasanoamuangae* Chittapun, Pholpunthin & Segers, 1999	PS, RF	NE, C, S	ORI (Known from Thailand, India and China)	11, 15, 24, 27, 39	
259	*Colurellasulcata* (Stenroos, 1898)	L, PS	NE, S	AFR, NEA, NEO, ORI, PAL	13, 14, 21, 24	
260	*Colurellatesselata* (Glascott, 1893)	PS	S	AFR, AUS, NEA, NEO, ORI, PAL	13, 24	
261	*Colurellauncinata* (Müller, 1773)	CA, L, P, PS, RF, RI, SW, TP	N, NE, C, S	AFR, AUS, NEA, NEO, ORI, PAL	6, 8, 10, 13, 14, 16, 18, 21, 23, 24, 25, 27, 39	
262	*Colurellauncinatabicuspidata* (Ehrenberg, 1830)	CA, L, P, RI	NE	AFR, AUS, NEA, NEO, ORI, PAL	4	Syn.: Colurellauncinata(Müller)f.bicuspidata (Ehrenberg) (Ref.4,16)
263	*Lepadellaacuminata* (Ehrenberg, 1834)	FF, L, P, PS, RE, RF, TP	NE, C, S	AFR, ANT, AUS, NEA, NEO, ORI, PAC, PAL	1, 3, 4, 11, 14, 18, 21, 22, 24, 25, 27	
264	*Lepadellaakrobeles* Myers, 1934	CA, P, RF, SW	NE	AUS, NEA, ORI	12, 18, 39	
265	*Lepadellaamphitropis* Harring, 1916	no information	NE	AFR, AUS, NEA, NEO, ORI, PAL	21	
266	*Lepadellaapsicora* Myers, 1834	L, P, PS, RF, TP	NE, S	AFR, AUS, NEA, NEO, ORI	4, 6, 8, 14, 16, 18, 21, 24, 25, 39	
267	*Lepadellaapsida* Harring, 1916	L, PS, RF	NE, S	AFR, AUS, NEA, NEO, ORI, PAL	4, 6, 8, 11, 14, 24, 39	
268	*Lepadellabenjamini* Harring, 1916	L	NE	AFR, AUS, NEA, NEO, ORI, PAL	14, 18	
269	*Lepadellabiloba* Hauer, 1958	L, RE	NE, S	AFR, AUS, NEO, ORI, PAL	4, 6, 8, 14, 18	
270	*Lepadellacostatoides* Segers, 1992	CA, L, P, PS, RF, SW, TP	N, NE, C, S	AFR, AUS, NEA, NEO, ORI, PAL	4, 10, 14, 18, 21, 23, 24, 25, 39	
271	*Lepadellacristata* (Rousselet, 1893)	L, PS	NE, S	AFR, NEA, NEO, ORI, PAL	13, 14	
272	*Lepadelladactyliseta* (Stenroos, 1898)	L, P, PS, RF, TP	NE, C, S	AFR, AUS, NEA, NEO, ORI, PAL	4, 6, 8, 13, 25, 39, 40	Syn.: *Lepadellavoigti* Hauer, 1931 (Ref.39)
273	*Lepadelladesmeti* Segers & Chittapun, 2001	PS	S	NEO, ORI, PAC	15, 24	
274	*Lepadelladiscoidea* Segers, 1993	CA, L, P, PS, RE, RF, SW, TP	N, NE, C, S	AFR, AUS, ORI	4, 10, 11, 13, 14, 16, 18, 23, 25, 39	
275	*Lepadellaehrenbergii* (Perty, 1850)	CA, L, P, PS, RE, SW	N, NE, C, S	AFR, AUS, NEA, NEO, ORI, PAL	4, 10, 14, 16, 18, 21, 23, 24	Incl.: *Lepadellaehrenbergi* (Perty, 1850) (Ref.4,10,14,16,18,21,23,24)
276	*Lepadellaelliptica* Wulfert, 1939	FF	NE, C	ANT, AUS, NEO, ORI, PAL	3, 21	
277	*Lepadellaelongata* Koste, 1992	L	NE	NEO, ORI, PAC	12, 14, 18, 21	Incl.: Lepadellacf.elongata Koste, 1992 (Ref.14)
278	*Lepadellaeurysterna* Myers, 1942	L, PS	NE, S	AFR, NEA, ORI, PAL	14, 18, 24	
279	*Lepadellaheterostyla* (Murray, 1913)	L	NE, S	AFR, AUS, NEA, NEO, ORI, PAL	6, 8, 18, 21	
280	*Lepadellalatusinus* (Hilgendorf, 1899)	CA, L, P, PS, RE, RF	N, NE, C, S	AFR, AUS, NEA, NEO, ORI, PAL	2, 4, 6, 8, 10, 14, 24	
281	*Lepadellalindaui* Koste, 1981	L, RF	NE, C, S	AFR, AUS, NEO, ORI	6, 8, 14, 18, 21, 29	
282	*Lepadellaminoruoides* Koste & Robertson, 1983	L, PS	S	AFR, NEO, ORI	6, 8, 24	
283	*Lepadellamonodactyla* Bērziņš, 1960	L, PS	NE, C, S	AFR, AUS, NEA, NEO, ORI	2, 14, 16, 24	Syn.: Lepadellamonostylaf.caudata (Koste, 1972) (Ref.2)
284	*Lepadellaovalis* (Müller, 1786)	CA, FF, L, PS, RF, SW, TP	N, NE, C, S	AFR, AUS, NEA, NEO, ORI, PAC, PAL	2, 3, 4, 6, 8, 10, 11, 13, 14, 18, 21, 23, 24, 25, 27, 39	
285	*Lepadellapatella* (Müller, 1773)	CA, FF, L, P, PS, RE, RF, SW, TP	N, NE, C, S	AFR, ANT, AUS, NEA, NEO, ORI, PAC, PAL	2, 3, 4, 6, 8, 10, 11, 13, 14, 16, 18, 21, 23, 24, 25, 27, 39	
286	*Lepadellapunctata* Wulfert, 1939	PS	S	ORI, PAL	17	
287	*Lepadellaquadricarinata* (Stenroos, 1898)	L, PS, RF	N, NE, S	AFR, AUS, NEA, NEO, ORI, PAL	4, 6, 8, 10, 11, 13, 14, 18	
288	*Lepadellaquinquecostata* (Lucks, 1912)	CA, L	N, NE	AFR, AUS, NEA, NEO, ORI, PAC, PAL	10, 14, 18	
289	*Lepadellarhomboides* (Gosse, 1886)	CA, L, P, PS, RE, RF, RI, SW, TP	N, NE, C, S	AFR, AUS, NEA, NEO, ORI, PAC, PAL	4, 5, 6, 8, 10, 11, 13, 14, 16, 18, 21, 22, 23, 24, 25, 27, 39	
290	*Lepadellatriba* Myers, 1934	L, PS, RF, TP	NE, C, S	AFR, AUS, NEA, NEO, ORI, PAC, PAL	2, 6, 8, 11, 14, 18, 21, 24, 25, 39	
291	*Lepadellatriptera* (Ehrenberg, 1830)	L, PS, RF, RI	NE, S	AFR, ANT, AUS, NEA, NEO, ORI, PAC, PAL	4, 14, 18, 21, 24, 39	Incl.: Lepadellatripteraf.alata (Ref.14,18)
292	*Lepadellavandenbrandei* Gillard, 1952	CA, L, P, PS, TP	N, NE, S	AFR, AUS, ORI	4, 6, 8, 10, 13, 14, 16, 18, 21, 24, 25	
293	*Paracolurellaaemula* (Myers, 1934)	PS	S	NEA, NEO, ORI	17	
294	*Squatinellalamellaris* (Müller, 1786)	L, P, PS, RF	NE, S	AFR, AUS, NEA, NEO, ORI, PAC, PAL	4, 6, 8, 13, 14, 18, 24, 39	Syn.: *Squatinellamutica* (Ehrenberg) (Ref.13,24), Squatinellalamellaris(Müller)f.mutica (Ehrenberg) (Ref.4,14,18)
295	*Squatinellaleydigii* (Zacharias, 1886)	PS	S	AUS, NEA, NEO, ORI, PAL	11, 16	Incl.: Squatinellaleydigiif.longiseta (Ref.11,16)
	**Family Lindiidae**					
296	*Lindiatorulosa* Dujardin, 1841	PS	S	AFR, ANT, AUS, NEA, NEO, ORI, PAL	20	
	**Family Mytilinidae**					
297	*Lophocharissalpina* (Ehrenberg, 1834)	CA, L, P, RE, RF, RI, TP	N, NE, C	AFR, AUS, NEA, NEO, ORI, PAL	4, 10, 14, 18, 21, 25, 27, 39	
298	*Mytilinaacanthophora* Hauer, 1938	CA, L, P, RF	N, NE	AFR, AUS, NEO, ORI, PAL	4, 10, 14, 18	
299	*Mytilinabisulcata* (Lucks, 1912)	CA, FF, L, RF, SW, TP	N, NE, C	AFR, AUS, NEO, ORI, PAL	3, 10, 14, 18, 23, 25, 27, 39	
300	*Mytilinacompressa* (Gosse, 1851)	L, P	NE, S	AFR, NEO, ORI, PAL	4, 6, 8	
301	*Mytilinacrassipes* (Lucks, 1912)	RE	C	AUS, NEA, ORI, PAL	22	
302	*Mytilinamichelangellii* Reid & Turner, 1988	RE	C	AFR, NEO, ORI	22	Syn.: Mytilinaventralisf.diversicantha Wulfert, 1965 (Ref.22)
303	*Mytilinamucronata* (Müller, 1773)	RE	C	AFR, AUS, NEA, NEO, ORI, PAL	22	
304	*Mytilinatrigona* (Gosse, 1851)*	RF	NE	AUS, NEA, NEO, ORI, PAL	39	
305	*Mytilinaunguipes* (Lucks, 1912)	CA, L, RF, SW	N, NE, C	AFR, NEO, ORI, PAL	4, 10, 14, 18, 23, 27, 39	
306	*Mytilinaventralis* (Ehrenberg, 1830)	CA, FF, L, P, PS, RE, RF, RI, SW, TP	N, NE, C, S	AFR, AUS, NEA, NEO, ORI, PAC, PAL	3, 4, 10, 11, 13, 14, 18, 21, 22, 23, 24, 25, 27, 39, 40, 41	
	**Family Notommatidae**					
307	*Cephalodellaforficula* (Ehrenberg, 1830)	L, P, PS, RF, TP	NE, C, S	AUS, NEA, NEO, ORI, PAC, PAL	2, 4, 14, 21, 24, 25, 39	Syn.: *Cephalodellaforficulaforficula* (Ehrenberg, 1830) (Ref.2)
308	*Cephalodellagibba* (Ehrenberg, 1830)	L, P, PS, RF	NE, C, S	AFR, ANT, AUS, NEA, NEO, ORI, PAC, PAL	2, 4, 6, 8, 14, 16, 18, 20, 21, 24, 29, 39	
309	Cephalodellacf.hyalina Myers, 1924	PS	S	NEA, ORI, PAL	11	
310	*Cephalodellainnesi* Myers, 1924	PS, RF	C, S	NEA, ORI, PAL	11, 15, 16, 20, 24, 29	
311	*Cephalodellamegalocephala* (Glascott, 1893)	PS	S	ANT, AUS, NEA, NEO, ORI, PAC, PAL	15	
312	*Cephalodellamucronata* Myers, 1924	L, PS, SW	NE, C, S	AFR, AUS, NEA, NEO, ORI, PAL	13, 14, 23	
313	Cephalodellacf.pachyodon Wulfert, 1937	L	NE	ORI, PAL	4	
314	*Cephalodellaplicata* Myers, 1924	PS	S	AUS, NEA, ORI, PAL	15	
315	*Cephalodellasongkhlaensis* Segers & Pholpunthin, 1997	L	NE, S	ORI (Known from Thailand and Cambodia)	8, 21	
316	*Cephalodellatenuior* (Gosse, 1886)	L, PS	NE, S	ANT, AUS, NEA, ORI, PAL	14, 24	
317	*Cephalodellatenuiseta* (Burn, 1890)	RF	C	AUS, NEA, NEO, ORI, PAL	29	
318	*Cephalodellaventripes* (Dixon-Nuttall, 1901)	P	NE	AUS, NEA, NEO, ORI, PAC, PAL	4	
319	Eosphoracf.thoides Wulfert, 1935	P	NE	AFR, AUS, NEA, NEO, ORI, PAL	4	
320	*Monommataactices* Myers, 1930	L	NE, S	AUS, NEA, NEO, ORI, PAL	6, 8, 18	
321	*Monommatadentata* Wulfert, 1940	PS	S	AUS, NEA, ORI, PAC, PAL	16, 24	
322	*Monommatagrandis* Tessin, 1890	PS, RF	NE, S	AFR, AUS, NEA, NEO, ORI, PAL	11, 13, 16, 24, 39	Incl.: *Monommatagrande* Tessin, 1890 (Ref.24)
323	*Monommatalongiseta* (Müller, 1786)	PS, RF	NE, S	AFR, AUS, NEA, NEO, ORI, PAL	11, 13, 16, 39	
324	*Monommatamaculata* Harring & Myers, 1930	PS	S	AFR, AUS, NEA, NEO, ORI, PAC, PAL	13	
325	*Notommatacopeus* Ehrenberg, 1834	L, P, PS	NE, S	AFR, AUS, NEA, NEO, ORI, PAL	4, 6, 8, 14, 24	
326	*Notommatapachyura* (Gosse, 1886)	CA, L, PS, RF	N, NE, S	AFR, AUS, NEA, NEO, ORI, PAL	4, 10, 13, 14, 18, 21	Incl.: Notommatapachyuraf.spinosa (Ref.13)
327	*Notommatapseudocerberus* Beauchamp, 1908	L	S	AFR, AUS, NEA, NEO, ORI, PAC, PAL	6, 8	
328	*Notommatapygmaea* Harring & Myers, 1922	PS	S	NEA, ORI	11	
329	*Notommatasaccigera* Ehrenberg, 1830	PS	S	AFR, AUS, NEA, NEO, ORI, PAL	13, 16, 24	
330	*Taphrocampaannulosa* Gosse, 1851	PS	S	AFR, AUS, NEA, NEO, ORI, PAL	11, 24	
	**Family Scaridiidae**					
331	*Scaridiumbostjani* Daems & Dumont, 1974	CA, L, P, PS	N, NE, S	AFR, AUS, NEA, NEO, ORI, PAL	4, 6, 8, 10, 24	
332	*Scaridiumelegans* Segers & De Meester, 1994	L, PS, SW	NE, S	AFR, AUS, NEO, ORI	12, 13, 14	
333	*Scaridiumgrande* Segers, 1995	CA, L, PS	N, NE, S	AFR, ORI	10, 13, 14, 16	Incl.: *Scaridiumgrandis* Segers, 1995 (Ref.10,14)
334	*Scaridiumlongicauda* (Müller, 1786)	L, P, PS, RE, RF, RI, SW, TP	N, NE, C, S	AFR, ANT, AUS, NEA, NEO, ORI, PAC, PAL	4, 6, 8, 10, 11, 13, 14, 16, 18, 21, 23, 24, 25, 39, 40	Incl.: *Scaridiumlongicauda* (Ref.40)
	**Family Synchaetidae**					
335	*Ploesomahudsoni* (Imhof, 1891)	CA, L, P, RE, RI, SW	NE, C	NEA, NEO, ORI, PAL	4, 14, 18, 21, 23	
336	*Ploesomalenticulare* Herrick, 1885	L, SW	NE, C	AFR, AUS, NEA, NEO, ORI, PAL	14, 23	
337	*Polyarthraeuryptera* Wierzejski, 1891	RE	no information	NEA, ORI, PAL	3	
338	*Polyarthralongiremis* Carlin, 1943	P, RE	NE	AUS, NEA, NEO, ORI, PAL	4	
339	*Polyarthramajor* Burckhardt, 1900	L, P, RE, RF	N, NE	AUS, NEA, NEO, ORI, PAL	4, 10	
340	*Polyarthraminor* Voigt, 1904	L, PS	S	AUS, NEA, ORI, PAL	6, 8, 11, 16	
341	*Polyarthraremata* Skorikov, 1896	L	S	AUS, NEA, NEO, ORI, PAL	6, 8	
342	*Polyarthravulgaris* Carlin, 1943	CA, L, P, PS, RE, RF, RI, SW, TP	N, NE, C, S	AFR, AUS, NEA, NEO, ORI, PAC, PAL	1, 3, 4, 5, 6, 8, 10, 11, 13, 14, 18, 21, 22, 23, 24, 25, 27, 39	Incl.: Polyarthracf.vulgaris Carlin, 1943 (Ref.25)
343	*Synchaetalongipes* Gosse, 1887	P	NE	AFR, AUS, NEA, NEO, ORI, PAL	4	
344	*Synchaetapectinata* Ehrenberg, 1832	CA, L, P, RE, RI	N, NE	AFR, AUS, NEA, NEO, ORI, PAL	4, 10, 18	
345	*Synchaetastylata* Wierzejski, 1893	L, P, RE, RI	NE, C	AFR, AUS, NEA, NEO, ORI, PAL	4, 14, 18, 21, 22	
	**Family Testudinellidae**					
346	*Pompholyxcomplanata* Gosse, 1851	CA, FF, L, P, RE, RF, RI, SW, TP	N, NE, C	AFR, AUS, NEA, NEO, ORI, PAL	1, 3, 4, 5, 10, 18, 21, 22, 23, 25, 27, 36	
347	*Testudinellaahlstromi* Hauer, 1956	CA, L, PS, RF, TP	N, NE, S	AUS, NEA, NEO, ORI	10, 13, 14, 18, 21, 25, 39	Rare species, Syn.: *Testudinellaincisaahlstromi* (Hauer) (Ref.13)
348	*Testudinellaamphora* Hauer, 1938	L, PS	NE, S	AUS, NEO, ORI	13, 14, 18, 24	
349	*Testudinellabrevicaudata* Yamamoto, 1951	L, P, RF, TP	N, NE, S	AFR, ORI, PAL	4, 6, 8, 10, 14, 18, 25	
350	*Testudinellaemarginula* (Stenroos, 1898)	P, PS	NE, S	AFR, AUS, NEA, NEO, ORI, PAL	4, 18, 24	
351	*Testudinellagreeni* Koste, 1981	L, RF, TP	N, NE	AFR, AUS, NEO, ORI	10, 14, 18, 25, 39	Rare species
352	*Testudinellaincisa* (Ternetz, 1892)*	RF	C	AFR, AUS, NEA, NEO, ORI, PAL	40	
353	*Testudinellamucronata* (Gosse, 1886)	PS	S	AFR, AUS, NEA, NEO, ORI, PAL	13	
354	*Testudinellaparva* (Ternetz, 1892)	L, P, PS, RE, TP	NE, C, S	AFR, AUS, NEA, NEO, ORI, PAL	4, 13, 14, 16, 18, 21, 22, 25	Syn.: *Testudinellainsinuata* Hauer, 1938 (Ref.18); T.cf.insinuata Hauer, 1938 (Ref.14)
355	*Testudinellapatina* (Hermann, 1783)	CA, FF, IT, L, P, PS, RE, RF, RI, SW, TP	N, NE, C, S	AFR, AUS, NEA, NEO, ORI, PAC, PAL	3, 4, 5, 6, 8, 10, 11, 13, 14, 16, 18, 21, 22, 23, 24, 25, 27, 36, 39, 40, 41	Syn.: Testudinellapatina(Hermann)f.typica (Ref.4) Incl.: Testudinellapatinaf.intermedia (Ref.4,22)
356	*Testudinellatridentata* Smirnov, 1931	CA, L, P, PS, RF, TP	N, NE, C, S	AFR, AUS, NEA, NEO, ORI, PAL	4, 10, 13, 14, 16, 18, 21, 25, 27, 41	
357	*Testudinellawalkeri* Koste & Shiel, 1980	L, PS	NE, S	AUS, ORI	6, 8, 14, 18, 21	
	**Family Tetrasiphonidae**					
358	*Tetrasiphonhydrocora* Ehrenberg, 1840	PS	S	AFR, AUS, NEA, NEO, ORI, PAL	13	
	**Family Trichocercidae**					
359	*Trichocercaabilioi* Segers & Sarma, 1993	L	NE	AFR, NEO, ORI	14, 18	
360	*Trichocercabicristata* (Gosse, 1887)	FF, L, P, RF, SW, TP	N, NE, C	AFR, AUS, NEA, NEO, ORI, PAC, PAL	3, 4, 10, 14, 18, 21, 23, 25, 39	
361	*Trichocercabidens* (Lucks, 1912)	L, PS, SW, TP	NE, C, S	AFR, ANT, AUS, NEA, NEO, ORI, PAC, PAL	4, 14, 18, 21, 23, 24, 25	
362	*Trichocercabraziliensis* (Murray, 1913)	CA, L, P, PS, RE, RF, RI, SW, TP	N, NE, C, S	AFR, AUS, NEO, ORI	4, 10, 11, 13, 14, 16, 18, 21, 23, 24, 25, 27	Incl.: *Trichocercabrasiliensis* (Murray, 1913) (Ref.13)
363	*Trichocercacapucina* (Wierzejski & Zacharias, 1893)	CA, L, P, PS, RE, RF, SW, TP	N, NE, C, S	AFR, AUS, NEA, NEO, ORI, PAL	4, 10, 14, 18, 21, 22, 23, 24, 25, 39	
364	*Trichocercachattoni* (Beauchamp, 1907)	P, PS, RE, TP	NE, C, S	AFR, AUS, NEO, ORI, PAL	4, 18, 21, 22, 24, 25	
365	*Trichocercacollaris* (Rousselet, 1896)	L, PS	NE, S	AFR, AUS, NEA, NEO, ORI, PAL	13, 14	
366	*Trichocercacylindrica* (Imhof, 1891)	FF, IT, L, P, RE, RI, SW, TP	NE, C, S	AFR, AUS, NEA, NEO, ORI, PAL	3, 4, 6, 8, 14, 18, 21, 22, 23, 25	
367	*Trichocercadixonnuttalli* (Jennings, 1903)	L, TP	NE, S	AFR, AUS, NEA, NEO, ORI, PAL	8, 14, 25	Syn.: *Trichocercainermis* (Linder, 1904) (Ref.14,25)
368	*Trichocercaelongata* (Gosse, 1886)	L, RE	NE, C	AFR, AUS, NEA, NEO, ORI, PAL	4, 14, 18, 22	
369	*Trichocercaflagellata* Hauer, 1937	CA, L, P, PS, RE, SW, TP	N, NE, C, S	AFR, AUS, NEO, ORI, PAL	4, 6, 8, 10, 13, 14, 18, 23, 24, 25	
370	*Trichocercahollaerti* De Smet, 1990	L, PS, TP	NE, S	AFR, NEO, ORI, PAC	4, 6, 8, 13, 14, 18, 24, 25	Rare species
371	*Trichocercainsignis* (Herrick, 1885)	L, P, PS, RF, TP	N, NE, S	AFR, AUS, NEA, NEO, ORI, PAC, PAL	4, 6, 8, 10, 13, 14, 18, 21, 25	
372	*Trichocercainsulana* (Hauer, 1937)	L, PS, RF, TP	NE, C, S	AFR, AUS, NEA, NEO, ORI, PAC, PAL	4, 11, 14, 21, 24, 25, 27, 29	Syn.: *Trichocercamontana* Hauer, 1956 (Ref.14)
373	*Trichocercalongiseta* (Schrank, 1802)	L, RE, TP	NE, C	AFR, AUS, NEA, NEO, ORI, PAL	14, 18, 21, 22, 25	
374	*Trichocercamus* Hauer, 1938	PS, SW	C, S	AUS, NEA, NEO, ORI, PAL	23, 24	
375	*Trichocercaobtusidens* (Olofsson, 1918)	L, RI	NE, S	NEA, NEO, ORI, PAC, PAL	4, 6, 8	Syn.: *Trichocercarelicta* Donner, 1950 (Ref.4,6,8)
376	*Trichocercaorca* (Murray, 1913)	L, SW	NE	AUS, ORI	14	
377	*Trichocercaporcellus* (Gosse, 1851)	L	NE	AFR, AUS, NEA, NEO, ORI, PAL	12, 14, 18	
378	*Trichocercapusilla* (Jennings, 1903)	CA, FF, L, P, PS, RE, RF, RI, SW, TP	N, NE, C, S	AFR, AUS, NEA, NEO, ORI, PAC, PAL	1, 3, 4, 5, 10, 11, 14, 18, 20, 21, 22, 23, 24, 25, 27, 39	
379	*Trichocercarosea* (Stenroos, 1898)	L	NE	AUS, NEA, NEO, ORI, PAL	14	
380	*Trichocercarousseleti* (Voigt, 1902)	L, P, RI	NE	AFR, AUS, NEA, ORI, PAL	4	
381	*Trichocercaruttneri* Donner, 1953	L, P, PS, RE	NE, S	AFR, AUS, NEA, NEO, ORI, PAL	4, 6, 8, 14, 24	
382	*Trichocercascipio* (Gosse, 1886)	L, PS	NE, S	AFR, AUS, NEA, NEO, ORI, PAC, PAL	14, 16, 18	Syn.: *Trichocercajenningsi* Voigt, 1957 (Ref.14,16)
383	*Trichocercasiamensis* Segers & Pholpunthin, 1997	L, PS	NE, S	NEA, NEO, ORI	8, 14, 16, 21	
384	*Trichocercasimilis* (Wierzejski, 1893)	CA, IT, L, P, PS, RE, RF, RI, SW, TP	N, NE, C, S	AFR, AUS, NEA, NEO, ORI, PAC, PAL	3, 4, 5, 6, 8, 10, 11, 14, 18, 21, 22, 23, 24, 25, 39, 40, 41	
385	*Trichocercasimilisgrandis* Hauer, 1965	PS, RF	C, S	AFR, AUS, NEO, ORI	13, 16, 27	Syn.: Trichocercasimilis(Weirzejski)f.grandis Hauer (Ref.13,16)
386	*Trichocercasimoneae* De Smet, 1990	L, TP	NE	AFR, NEO, ORI, PAC, PAL	14, 18, 25	Incl.: *Trichocercasimonei* De Smet, 1989 (Ref.14,18,25)
387	*Trichocercastylata* (Gosse, 1851)	L, P, SW, TP	NE, C	AFR, AUS, NEA, NEO, ORI, PAC, PAL	4, 14, 18, 23, 25	
388	*Trichocercatenuidens* (Hauer, 1931)	P	NE	ORI, PAL	4	
389	*Trichocercatenuior* (Gosse, 1886)	FF, L, PS, RF, TP	N, NE, S	AFR, AUS, NEA, NEO, ORI, PAC, PAL	3, 10, 14, 15, 18, 20, 21, 24, 25, 39	
390	*Trichocercatigris* (Müller, 1786)	L, P, PS, RI	NE, S	AFR, ANT, AUS, NEA, NEO, ORI, PAC, PAL	4, 14, 21, 24	
391	*Trichocercavernalis* (Hauer, 1936)	L	NE	AUS, NEA, ORI, PAL	14	
392	*Trichocercavoluta* (Murray, 1913)	L, PS, RE	NE, S	NEO, ORI	4, 6, 8, 13, 14, 18	Syn.: *Trichocercatropis* Hauer, 1937 (Ref.4,6,8,13,14,18)
393	*Trichocercaweberi* (Jennings, 1903)	L, PS, RF	NE, S	AFR, AUS, NEA, NEO, ORI, PAC, PAL	14, 18, 21, 24, 39	
	**Family Trichotriidae**					
394	*Macrochaetuscollinsii* (Gosse, 1867)	CA, L, P, PS, RE, RF, SW, TP	N, NE, C, S	AFR, AUS, NEA, NEO, ORI, PAL	2, 4, 10, 11, 13, 14, 16, 18, 21, 23, 24, 25	Incl.: *Macrochaetuscollinsi* (Gosse, 1867) (Ref.2,4,10,13,14,16,18,21,23,24,25)
395	*Macrochaetusdanneelae* Koste & Shiel, 1983	L, RF, TP	N, NE	AUS, ORI	10, 14, 18, 25, 39	Rare species, Incl.: *Macrochaetusdanneeli* Koste & Shiel, 1983 (Ref.10,14,18,25)
396	*Macrochaetuslongipes* Myers, 1934	CA, L, P, RF, SW, TP	N, NE, C	AFR, NEA, NEO, ORI, PAL	4, 10, 14, 18, 23, 25	
397	*Macrochaetussericus* (Thorpe, 1893)	L, P, TP	NE, S	AFR, NEA, NEO, ORI, PAL	4, 6, 8, 14, 18, 21, 25	
398	*Macrochaetussubquadratus* Perty, 1850	L, PS, SW	NE, C, S	AFR, AUS, NEA, NEO, ORI, PAL	13, 14, 18, 23	
399	*Trichotriatetractis* (Ehrenberg, 1830)	CA, L, P, PS, RE, RF, RI, SW, TP	N, NE, C, S	AFR, AUS, NEA, NEO, ORI, PAC, PAL	4, 6, 8, 10, 13, 14, 16, 18, 21, 22, 23, 25, 36, 39, 40, 41	
400	*Wolgaspinifera* (Western, 1894)	TP	NE	AFR, AUS, NEA, NEO, ORI, PAL	12, 18, 25	
	**Family Trochosphaeridae**					
401	*Filiniabrachiata* (Rousselet, 1901)	RE	C	AFR, AUS, NEA, ORI, PAL	3, 22	
402	*Filiniacamasecla* Myers, 1938	CA, L, P, RE, RF, RI, SW, TP	N, NE, C	ORI (Known from Thailand, Cambodia and India)	4, 5, 10, 14, 18, 21, 22, 23, 25, 27, 29, 41	
403	*Filinialongiseta* (Ehrenberg, 1834)	CA, IT, L, P, PS, RE, RF, RI, SW, TP	N, NE, C, S	AFR, AUS, NEA, NEO, ORI, PAL	1, 3, 4, 10, 11, 18, 21, 22, 23, 24, 25, 27, 36, 40, 41	Incl.: Filinialongisetavar.limnetica (Ref.22)
404	*Filinianovaezealandiae* Shiel & Sanoamuang, 1993	L, P, PS, RE, RF, SW, TP	NE, C, S	AFR, AUS, NEA, ORI	11, 14, 18, 21, 22, 23, 25, 27, 29, 41	
405	*Filiniaopoliensis* (Zacharias, 1898)	CA, FF, L, P, PS, RE, RF, RI, SW, TP	N, NE, C, S	AFR, AUS, NEA, NEO, ORI, PAL	1, 3, 4, 5, 10, 11, 13, 14, 18, 21, 22, 23, 24, 25, 27, 36, 40, 41	Syn.: *Tetramastixopoliensis* Zacharias, 1898 (Ref.1)
406	*Filiniapejleri* Hutchinson, 1964	CA, L, P, RE, RF, RI	N, NE	AFR, ANT, AUS, NEO, ORI, PAL	4, 10	
407	*Filiniasaltator* (Gosse, 1886)	P, RF	N, NE	AFR, NEO, ORI, PAL	4, 10, 18	
408	*Filiniaterminalis* (Plate, 1886)	FF, L, RE, RF	NE, C	AFR, AUS, NEA, NEO, ORI, PAL	3, 4, 40	
409	*Trochosphaeraaequatorialis* Semper, 1872	CA, FF, L, P, RE, RF, SW	N, NE, C	AUS, NEA, NEO, ORI, PAL	3, 4, 10, 18, 27, 36, 40, 41	

**Table 3.** Jaccard similarity index among six regions in Thailand. Bold numbers indicated the highest value. Regional distribution abbreviations are as defined in Table 2.

**Regions**	**NE**	**C**	**E**	**W**	**S**
**N**	0.45	0.44	0.23	0.07	0.33
**NE**		0.48	0.12	0.03	**0.53**
**C**			0.16	0.05	0.42
**E**				0.21	0.12
**W**					0.04

**Table 4.** Jaccard similarity index among habitat types. Bold numbers indicate the highest similarity values. Habitat type abbreviations are as described in Table 2.

**Habitat types**	**D**	**FF**	**IT**	**L**	**M**	**P**	**PS**	**RE**	**RF**	**RI**	**SW**	**TP**
**CA**	0.13	0.20	0.06	0.38	0.25	**0.53**	0.35	0.44	0.51	0.44	0.50	0.46
**D**		0.10	0.00	0.06	0.33	0.08	0.07	0.12	0.09	0.12	0.11	0.11
**FF**			0.05	0.14	0.16	0.21	0.13	0.24	0.22	0.16	0.24	0.23
**IT**				0.03	0.10	0.05	0.04	0.07	0.05	0.11	0.07	0.07
**L**					0.12	0.48	0.43	0.35	0.40	0.26	0.39	0.40
**M**						0.17	0.14	0.19	0.19	0.27	0.23	0.18
**P**							0.39	0.47	0.50	0.36	0.50	0.50
**PS**								0.30	0.44	0.25	0.36	0.40
**RE**									0.39	0.43	0.47	0.45
**RF**										0.33	0.43	**0.53**
**RI**											0.38	0.38
**SW**												0.46
